# ﻿*Erythrina* L. (Phaseoleae, Papilionoideae, Leguminosae) of Brazil: an updated nomenclatural treatment with notes on etymology and vernacular names

**DOI:** 10.3897/phytokeys.232.101105

**Published:** 2023-09-04

**Authors:** Ramon Guedes-Oliveira, Ana Paula Fortuna-Perez, Leandro Cardoso Pederneiras, Vidal de Freitas Mansano

**Affiliations:** 1 Programa de Pós-graduação em Botânica, Escola Nacional de Botânica Tropical (ENBT), Instituto de Pesquisas Jardim Botânico do Rio de Janeiro (JBRJ), 22460-036, Horto, Rio de Janeiro, State of Rio de Janeiro, Brazil Programa de Pós-graduação em Botânica, Escola Nacional de Botânica Tropical (ENBT), Instituto de Pesquisas Jardim Botânico do Rio de Janeiro (JBRJ) Rio de Janeiro Brazil; 2 Departamento de Biodiversidade e Bioestatística, Instituto de Biociências de Botucatu, (IBB), Universidade Estadual Paulista (UNESP), 18618–970, Botucatu, State of São Paulo, Brazil Universidade Estadual Paulista (UNESP) Botucatu Brazil; 3 Diretoria de Pesquisas (Dipeq), Instituto de Pesquisas Jardim Botânico do Rio de Janeiro (JBRJ), 22460-030, Jardim Botânico, Rio de Janeiro, State of Rio de Janeiro, Brazil Diretoria de Pesquisas (Dipeq), Instituto de Pesquisas Jardim Botânico do Rio de Janeiro (JBRJ) Rio de Janeiro Brazil

**Keywords:** Fabaceae, legumes, Linnaeus, nomenclature, papilionoid legume, South America, Vellozo

## Abstract

*Erythrina* L. is a genus that comprises ca. 120 to 130 species distributed throughout the tropics and subtropics of the world. Linnaeus established the genus in Genera Plantarum (1737) and the first binomial name given to a Brazilian *Erythrina* was *E.crista-galli* L., described by himself in Mantissa Plantarum (1767). Vellozo proposed in Florae Fluminensis (1790–1881) the first treatment of the genus in Brazil, where he treated three species from the states of Rio de Janeiro and São Paulo. Martins and Tozzi proposed the most recent treatment in 2018, where the authors recognized 11 valid names and presented three new synonyms. Despite extensive efforts already made in the genus, previous works did not treat all names related to the valid ones for Brazilian *Erythrina*. The present work is the most comprehensive and up-to-date nomenclatural treatment for the genus in Brazil, covering all 84 related names found on digital nomenclatural databases. Here we analyze 64 protologues, update typification statuses, propose five new synonyms, 13 new lectotypes (11 first-step, two second-step) and one neotype, linking all protologues and type specimens with their corresponding available digital sources, and make additional notes on etymology and vernacular names.

## ﻿Introduction

*Erythrina* L. (Phaseoleae, Papilionoideae, Leguminosae) is a genus that comprises ca. 120 to 130 species (Du Puy et al. 2002; Schrire 2005) distributed throughout the tropics and subtropics of the world in a wide range of habitats. The species can be found in arid tropical deserts or lowland alluvial vegetations to montane forests beyond 3 000 m of altitude, varying from rhizomatous perennial subshrubs to trees measuring more than 40 m high (Neill 1988).

Linnaeus established the genus in Genera Plantarum (Linnaeus 1737), expanded later in Species Plantarum (Linnaeus 1753), where he described three species (*E.herbacea* L., *E.corallodendrum* L., *E.piscipula* L.) and two varieties (E.corallodendrumvar.occidentalis L., E.corallodendrumvar.orientalis L.) based on plants mentioned earlier by other authors before the establishment of Linnean binomials. The type species of the genus was designated by Walpers (1853) as *E.herbacea*. The first binomial name given to a Brazilian *Erythrina* was *E.crista-galli* L., described by Linnaeus in Mantissa Plantarum (Linnaeus 1767).

The first treatment of the genus in Brazil was proposed by Vellozo’s Florae Fluminensis, which was done in 1790 but the text was only partially published in 1829, the plates in 1831, and the complete text in 1881 (Vellozo 1829, 1831, 1881; Carauta 1969, 1973). In this work, the author treated three species from the states of Rio de Janeiro and São Paulo: *E.corallodendrum* (which was considered to be a mention of *E.corallodendrum* L. rather than a new publication of this name), *E.verna* Vell., and *E.mediterranea* Vell.

In 1859, Bentham proposed a treatment of Brazilian *Erythrina* in Martius’ Flora Brasiliensis, where he published two new names (*E.falcata* Benth. and *E.mulungu* Mart. ex Benth.), mentioned five others already validly published (*E.corallodendrum* L. [misspelled as *corallodendron*], *E.crista-galli* L., *E.glauca* Willd., *E.reticulata* C.Presl., *E.velutina* Willd.), and one as a doubtful species (*E.nervosa* DC.).

The subsequent treatment of Brazilian *Erythrina* was proposed by Krukoff (1938), where he published two new names and a form (*E.amazonica* Krukoff, *E.similis* Krukoff and E.velutinaf.aurantiaca (Ridl.) Krukoff), mentioned ten others already validly published (*E.crista-galli*, *E.dominguezii* Hassl., *E.falcata*, *E.flammea* Herzog, *E.glauca* Willd., *E.poeppigiana* (Walp.) O.F.Cook, *E.speciosa* Andrews, *E.ulei* Harms, *E.velutina* Willd., *E.verna*), and three other names as doubtful and unplaced (*E.mediterranea*, *E.nervosa*, *E.secundiflora* Brot.). Krukoff reduced *E.mulungu* and *E.reticulata* to synonyms of *E.verna* and *E.speciosa*, respectively. *E.corallodendrum* was treated as a species that occurs only in Jamaica and Haiti, not Brazil as Bentham (1859) believed.

The most comprehensive revision of the genus to this day was proposed by Krukoff and Barneby (1974), who recognized 11 valid names for Brazilian *Erythrina* (*E.amazonica*, *E.crista-galli*, *E.dominguezii*, *E.falcata*, *E.fusca* Lour., *E.poeppigiana*, *E.similis*, *E.speciosa*, *E.ulei*, *E.velutina*, *E.verna*), published a new hybrid name (E.×fluminensis Barneby & Krukoff), maintained two names as doubtful and unplaced (*E.mediterranea*, *E.secundiflora*), and excluded *E.nervosa* as a synonym of *Callichlamyslatifolia* (A.Rich) K.Schum. (Bignoniaceae). The authors also considered the previously accepted *E.glauca* as synonym of *E.fusca*, and *E.flammea* as synonym of *E.verna*.

The most recent treatment of Brazilian *Erythrina* was proposed by Martins (2014) in a PhD thesis. In 2015, the author published a taxonomic treatment of the genus in Flora e Funga do Brasil (Martins 2023). The nomenclatural treatment was published later in Martins and Tozzi (2018), where the authors also recognized 11 valid names (*E.amazonica*, *E.crista-galli*, *E.falcata*, *E.fusca*, *E.mulungu*, *E.poeppigiana*, *E.similis*, *E.speciosa*, *E.ulei*, *E.velutina*, *E.verna*), designated 12 lectotypes and one epitype, and proposed three new synonyms: the previously accepted *E.dominguezii* as *E.mulungu* (which had been considered a synonym of *E.verna* since Krukoff [1938]), E.speciosavar.rosea N.F.Mattos as *E.speciosa*, and E.velutinaf.aurantiaca as *E.velutina*.

However, Martins and Tozzi (2018) did not cover all names related to Brazilian *Erythrina* species. We here present the most comprehensive and up-to-date nomenclatural treatment for Brazilian *Erythrina*, covering all related names found on digital nomenclatural databases. We update typification statuses, propose new synonyms, designate lectotypes and neotypes, linking all protologues and type specimens with its corresponding digital sources when available. We also provide additional notes on etymology and vernacular names for each accepted species.

## ﻿Methods

Scientific names of *Erythrina* L. were collected from the following digital databases: Global Biodiversity Information Facility (GBIF), International Legume Database & Information Service (ILDIS), International Plant Names Index (IPNI), Legume Data Portal, Plants of the World Online, The Plant List, w3Tropicos, World Flora Online. Original protologues were accessed through the following digital libraries and databases: Biblioteca Digital Real Jardín Botánico, Biblioteca Nacional Digital Brasil, Bielefeld University Library, Biodiversity Heritage Library, Flora Brasiliensis CRIA, Google Books, Google Scholar, Hathi Trust Digital Library, JSTOR Global Plants, Naturalis Biodiversity Center Library, in addition to the more recent articles published in peer-reviewed journals. The authorship and publication dates of protologues were confirmed through the Taxonomic Literature II (TL-2) and the Hunt Institute for Botanical Documentation (BPH) digital databases. The type specimens were analyzed in person only at the herbaria R and RB, the rest of them through digitalized images from the following herbaria: A, B, BAF, BM, BR, E, F, G, GH, HBG, HUEFS, IAN, K, L, LIL, LINN, LP, M, MEL, MG, MO, MPU, NAP, NY, P, S, SI, SP, TCD, U, US, VEN, W and Z, the acronyms following Thiers (continuously updated). Herbarium data were collected through the following digital databases and virtual herbaria: BioPortal, Conservatoire et Jardin botaniques Genève, Field Museum of Natural History, Harvard University Herbaria, Herbarium Berolinense, JABOT, JACQ, JSTOR Global Plants, Kew Royal Botanic Gardens, Meise Botanic Garden, Muséum National d’Histoire Naturelle, Reflora, Smithsonian National Museum of Natural History, speciesLink, Swedish Museum of Natural History, The Natural History Museum, The New York Botanical Garden, w3Tropicos, Zürich Herbaria. The validity of typifications was confirmed through the International Code of Nomenclature for algae, fungi, and plants (ICN) (Turland et al. 2018). Etymology information was based on the original protologues, and vernacular names on herbarium specimens’ labels and published studies of the genus.

## ﻿Results and discussion

From the nomenclatural search in digital databases, 84 names published in a total of 64 protologues were found and analyzed (Suppl. material 1). The nomenclatural revision resulted in the maintenance of 57 synonyms and confirmed the 11 currently valid names for Brazilian *Erythrina*. Here we propose five new synonyms, designate 11 first-step and two second-step lectotypes, and one neotype. Seven names remained as doubtful synonyms for several reasons explained in the species’ commentaries: E.crista-galliL.var.corallina N.F.Mattos, E.crista-galliL.var.laurifolia Tod., E.crista-galliL.var.speciosa Tod., *E.laurifolia* Tod. and *E.speciosa* Tod. as potential synonyms of *E.crista-galli* L.; and *E.atrosanguinea* Ridl. and *E.picta* Blanco as potential synonyms of *E.fusca* Lour. These two accepted species represent the Brazilian *Erythrina* with most names given throughout the years, from 1741 to 2010, being *E.crista-galli* one of the most cultivated *Erythrina* species around the world since the 18^th^ century, and *E.fusca* the most geographically widespread species in the genus (Krukoff and Barneby 1974). Moreover, five names remained unplaced (*E.indicasensu* R.Vig., *E.mediterranea* Vell., *E.moelebei* Vieill. ex Guillaumin & Beauvis., *E.secundiflora* Brot., *E.velutina* Jacq.) and eight were excluded (*Corallodendronnervosum* (DC.) Kuntze, *E.adansonii* hort. ex Colla, *E.argentea* Blume ex Miq., *E.compacta* W.Bull, *E.compacta* W.Bull ex K.Koch, *E.corallodendrum* Vell., E.fuscaLour.var.inermis Pulle, *E.nervosa* DC.) for several reasons explained in the corresponding section.

Up until the time of this publication, we were unable to access six of the original protologues: i) Index seminum horti regii botanici panormitani, regarding the names *E.laurifolia* and *E.speciosa* by Todaro (1860); ii) Nuovi generi e nuove specie di piante cultivate nel real Orto botanico di Palermo vol. 3, regarding *E.pulcherrima* by Todaro (1861); iii) Catálogo descriptivo de las maderas que se exhibieron en la exposición internacional de agricultura de 1910 in Anales de la Sociedad Rural Argentina, regarding *E. chacoënsis* and E.crista-gallivar.inermis by Spegazzini and Girola (1910); iv) Archives de botanique, mémoires vol. 6, regarding *E.indica* sensu R. Viguier; v) Loefgrenia; Comunicações Avulsas de Botânica vol. 21(1), regarding E.crista-gallivar.corallina by Mattos (1967); and vi) Loefgrenia; Comunicações Avulsas de Botânica vol. 71(3), regarding E.speciosavar.rosea by Mattos (1977).

### ﻿Nomenclatural treatment

#### ﻿ Erythrina

Taxon classificationPlantaeFabalesFabaceae

L., Gen. Pl.: 216. 1737; Sp. Pl. 2: 706. 1753.

74347752-908B-5EB3-BC16-9502EABBEFEE

##### Generitype.

(designated by Walpers 1853, pg. 145): *Erythrinaherbacea* L., Sp. Pl. 2: 706. 1753.

#### ﻿1. Erythrina amazonica

Taxon classificationPlantaeFabalesFabaceae

Krukoff, Brittonia 3: 270. 1938.

79FD97F6-1045-5951-AC35-916182A3F3A7

Fig. 1

##### Type material.

**Brazil. Amazonas**: Basin of Rio Jurua; Near mouth of Rio Embira (tributary of Rio Tarauaca), 8 June 1933, *Krukoff 4707* (holotype: NY [NY00007986, sheet I; NY00007987, sheet II]; isotypes: A [A00066284], G [G00365285, two sheets], K [K000502766, K000502767]).

##### Notes.

**Figure 1.**
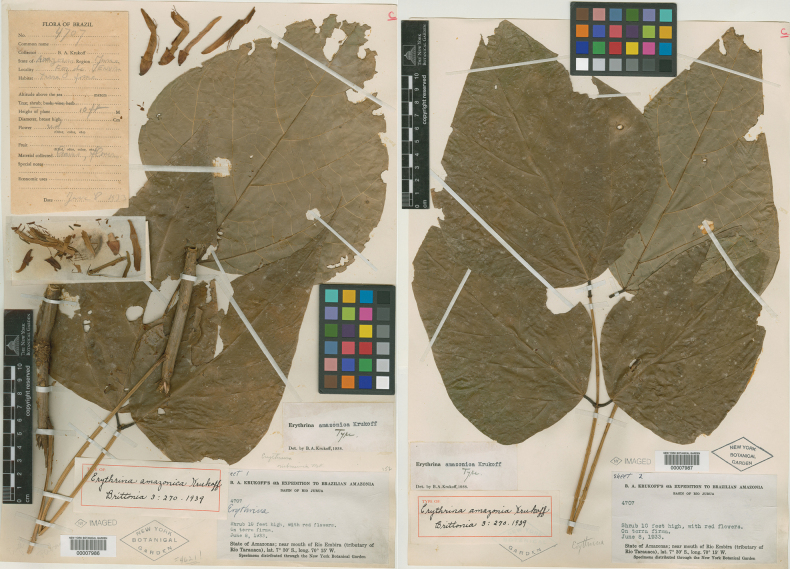
Holotype of *Erythrinaamazonica*Krukoff (1938: 270). Source: William and Lynda Steere Herbarium (NY) – The New York Botanical Garden via C. V. Starr Virtual Herbarium, NY00007986, NY00007987.

There are no nomenclature issues with *E.amazonica*, as the name was validly published and its type specimen was correctly cited (Fig. 1). However, all databases and studies of the genus state that the protologue was published in 1939. Still, according to the journal’s website, the publication date is October 1938.

##### Etymology.

The specific epithet “*amazonica*” is derived from Latin, meaning “from the Amazon”, and it was chosen due to the species being native to the Amazon Forest domain.

##### Vernacular names.

According to herbaria labels, *E.amazonica* is generally known as “mulungu” in Brazil, and also “açacurana” (and spelling variations) or “mulungu-de-espinho” in the state of **Maranhão**, and “açacurana” (and spelling variations) or “tento” in **Pará**.

#### ﻿2. Erythrina crista-galli

Taxon classificationPlantaeFabalesFabaceae

L., Mant. Pl.: 99. 1767.

D770F2DC-1F7E-53BE-986A-4A0BA4F6A0DF

Fig. 2

≡ Micropteryx crista-galli (L.) Walp., in Duchassaing and Walpers, Linnaea 23(=7): 740. 1851.
≡ Corallodendron crista-galli (L.) Kuntze, Revis. Gen. Pl. 1: 172. 1891.
= Erythrina laurifolia Jacq., Observ. Bot. 3: 1. 1768. Type: Argentina. s.loc., ex hort., s.d., s.leg., s.n. (lectotype, designated by Lozano and Zapater 2010, pg. 181: illustration in Jacquin 1768, tab. 51). (1)
= Erythrina graefferi hort. ex Tineo, Cat. Pl. Hort. Panorm.: 278. 1827. Type: Italy. Campania: “ex H. R. Cas. [Parco Reale – Reggia di Caserta]”, ex hort., s.d., *Gussone s.n.* (neotype, designated by Del Guacchio et al. 2019, pg. 119: NAP [image seen]). (2)
= Erythrina fasciculata Benth., Linnaea 22(=6): 517. 1849. Type: Brazil. Minas Gerais: “Ad Caldas”, [10 February 1846?], *Regnell I.73* (lectotype, designated by Lozano and Zapater 2010, pg. 183: K [K000674145, sheet I; K000930947, sheet II]; isolectotypes: K [K000930957], MEL [MEL77238], NY [NY01058578], P [P02934611], US [US02338870]). Residual syntype: Brazil. Goiás: “ad Fazenda do Salido ad Rios”, s.d., *Pohl 763*, BR (BR0000013057763), K (K000930946), M (M0240562, M0240563), NY (NY00007993), W (W0027158, W0027159). (3)
= Erythrina pulcherrima Tod., Nuov. Gen. Sp. 3: 70. 1861; Ann. Sci. Nat., Bot. 4(20): 307. 1863. Type: Italy. Sicily: “Jamdudum in horto panormitano [Orto Botanico di Palermo] culta et ut videtur ex Aegypto in Siciliam advecta”, ex hort., s.d., s.leg., s.n. (lectotype, designated by Martins and Tozzi 2018, pg. 398: illustration in Todaro 1876, tab. XI). (4)
= Erythrina crista-galli var. hasskarlii Backer, Voorl. Schoolfl. Java: 87. 1908. Type: Indonesia. West Java: “Cult. in Hort. Bog. [Kebun Raya Bogor]”, ex hort., [1904?], s.leg., s.n. (lectotype, designated here: L [L.1951408]; isolectotypes: L [L.1951407, L.1951409]). (5)
= Erythrina crista-galli var. leucochlora Lombardo, Flora arborea y arborescente del Uruguay: 69. 1964. Type: Uruguay. Treinta y Tres: “a orillas del río Cebollatí en “La charqueada””, s.d., s.leg., s.n. (lectotype, designated here: illustration in Lombardo 1964, tab. 66 [as E.cristagalli]). (6)
= Erythrina crista-galli var. longiflora Zapater & Lozano, in Lozano and Zapater, Darwiniana 48(2): 185. 2010. Type: Argentina. Salta: “Dpto. Gral. Güemes, ruta 9 en autopista de acceso a Salta, al frente de Usina Termo Andes”, 10 May 2008, *Zapater 2748b* (holotype: SI [SI022986]; isotype: MCNS [n.v.]). syn. nov. (7)

##### Type material.

**Brazil.** s.loc., s.d., *Vandelli s.n.* (lectotype, designated by Howard 1988, pg. 488: LINN [LINN-HL888-4]).

##### Notes.

**Figure 2.**
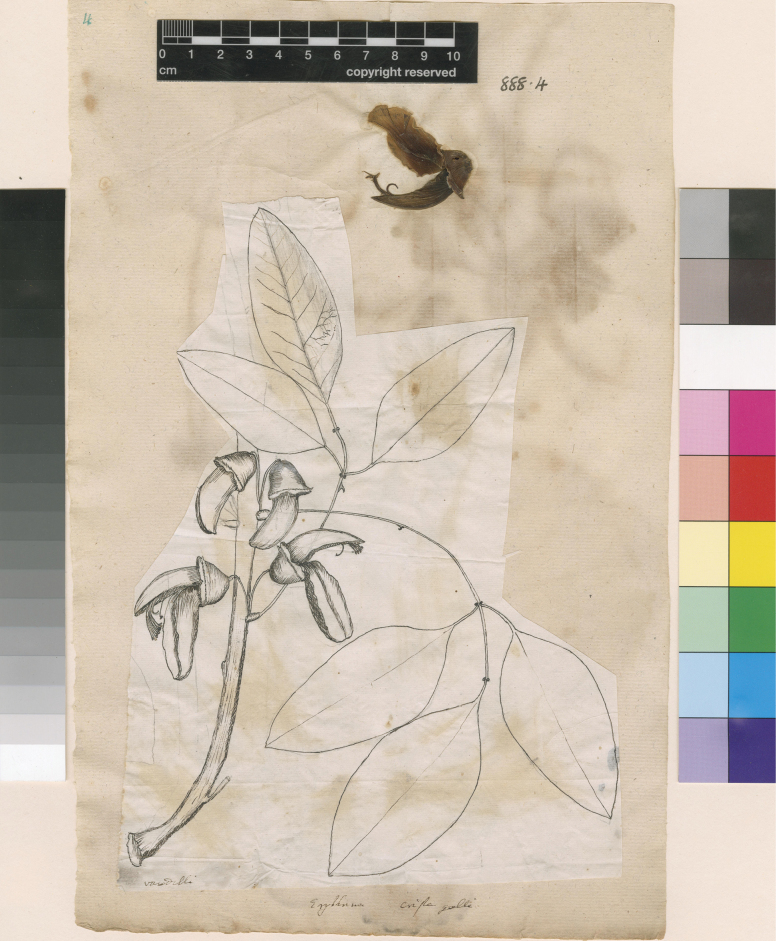
Lectotype of *Erythrinacrista-galli* L. (1767: 99), designated by Howard (1988: 488). Source: Linnean Society of London Herbarium (LINN) via JSTOR Global Plants, LINN-HL888-4.

The first known name for the species was published in Linnaeus (1767), who described it from a specimen collected by Vandelli in Brazil. Yet, he did not mention any collection number nor cite any herbarium. Krukoff and Barneby (1974) accessed an exsiccata from the herbarium LINN consisting of a drawing with the inscription “Vandelli” and a single flower that undoubtedly represents the species, considering this to be the original collection. However, this citation cannot be considered a lectotypification of the name, as the authors did not mention it as the type collection. In Flora of the Lesser Antilles, Howard (1988) correctly designated the LINN collection as the type specimen (Fig. 2). Walpers (1851, not 1850) published *Micropteryx* mentioning *E.crista-galli* as a synonym of *M.crista-galli*, but the genus was later synonymized into *Erythrina* in Engler and Prantl (1894). Kuntze (1891) published *Corallodendron* mentioning *E.crista-galli* as a synonym of *C.crista-galli*, but the genus was also synonymized under *Erythrina* in Engler and Prantl (1894).

(1) Jacquin (1768) published *E.laurifolia* with an illustration of a specimen from a botanical garden in Rome that is morphologically identical to *E.crista-galli*. Lozano and Zapater (2010) correctly designated this illustration as the lectotype, and the name has been synonymized under *E.crista-galli* since Lamarck (1786).

(2) Tineo (1827) published *E.grafferi*, a name used by gardeners at the Orto Botanico di Palermo (Italy), with a short description resembling *E.crista-galli*. Krukoff and Barneby (1974) doubted that it could represent *E.speciosa* Lamb. ex Andrews, but Del Guacchio et al. (2019) investigated its origin, confirmed its synonymity under *E.crista-galli* and correctly designated a neotype for the name.

(3) Bentham (1849) published *E.fasciculata* from Brazil, mentioning two collections that can be considered syntypes of the name, one from the state of Minas Gerais (*Regnell I.73*) and one from Goiás (*Pohl 763*). Krukoff and Barneby (1974) recognized those syntypes but did not designate a lectotype, which was then correctly designated by Lozano and Zapater (2010) as the collection from Regnell in herbarium K. Additional material: P (P02934612, photo of K000674145; P02934614, photo of W0027159).

(4) Todaro (1861) published *E.pulcherrima* based on a cultivated specimen at the Orto Botanico di Palermo (Italy). The original protologue could not be found online, but later publications by himself (Todaro 1863, 1876) also cited this name, including a well-made illustration in 1876 that even mentions the similarity with *E.crista-galli*. Martins and Tozzi (2018) correctly designated his illustration in 1876 as the lectotype, but wrongly considered this publication as the protologue of the name, which is the publication in 1861.

(5) Backer (1908) published the variety E.crista-gallivar.hasskarlii with a short description from cultivated plants on Java Island (Indonesia), but did not assign any type specimen. Three exsiccatae of a plant cultivated in the “*Hortus Bogoriensis*” [Kebun Raya Bogor] (Java) were found in herbarium L, with the inscription “Erythrinacrista-galli Linn. var. hasskarlii Backer”, that could represent the original material. One of them was designated here as the presumed lectotype, and the name has been synonymized under *E.crista-galli* since Krukoff and Barneby (1974).

(6) Lombardo (1964) published the variety E.crista-gallivar.leucochlora from Uruguay with a short description, but did not assign any type specimen. As there are no evident characters that support this variety besides the white flowers, which is a common mutation found in cultivated *Erythrina* species (Guedes-Oliveira et al. manuscript in preparation), the name has been synonymized under *E.crista-galli* since Krukoff and Barneby (1974), and Lombardo’s illustration in the same publication was designated here as lectotype.

(7) Lozano and Zapater (2010) published the variety E.crista-gallivar.longiflora from Argentina with a full description, an illustration and mentioning the type specimens. However, as there are no evident characters that support this variety besides the size of leaves and flowers, which is a character with well-documented morphological plasticity in *Erythrina* species (Guedes-Oliveira et al. manuscript in preparation), the name was here synonymized under *E.crista-galli*.

##### Etymology.

The specific epithet “*crista-gallicrista*” is derived from Latin, meaning “the crest of the rooster”, and it was presumably chosen after the usual association of the corolla with the shape and color of chicken combs.

##### Vernacular names.

*E.crista-galli* has a variety of vernacular names in many regions where it occurs, either native or introduced and cultivated. According to herbaria labels and Carvalho (2006), the species is generally known as “corticeira” in Brazil, and also “corticeira-de-beira-do-rio”, “corticeira-do-brejo”, “crista-de-galo”, “manequeira”, “mulungu”, “mulungu-de-espinho” or “suinã” (and spelling variations) in the state of **Mato Grosso do Sul**; “crista-de-galo”, “moxoqueiro-do-brejo”, “samaúva” or “samauveira” in **Minas Gerais**; “cortiça”, “corticeira-do-banhado”, “corticeira-do-brejo”, “crista-de-galo”, “flor-de-coral”, “mulungu”, “sananduva” or “suinã” (and spelling variations) in **Paraná**; “crista-de-galo” in **Rio de Janeiro**; “corticeira-do-banhado”, “marrequinha” or “sananduva” in **Rio Grande do Sul**, where it is also the motive for the name of the municipality of Sananduva; “corticeira-do-banhado”, “corticeira-bico-de-papagaio”, “corticeira-do-brejo”, “flor-de-coral” or “marrequeira” in **Santa Catarina**; and “crista-de-galo”, “flor-de-coral”, “maçã-de-cobra”, “muchocho” (and spelling variations), “mulungú”, “patinha”, “samauveira”, “sananduva” or “suinã” (and spelling variations) in **São Paulo**.

#### Doubtful synonyms of *Erythrinacrista-galli*

##### ﻿ Erythrina crista-galli var. laurifolia

Taxon classificationPlantaeFabalesFabaceae

Tod., Index. Sem. Panorm. 1861: 32. 1862

8DF8C18D-3EE5-5AF2-87C3-7E0604D87726

≡ Erythrina laurifolia Tod., Index. Sem. Panorm. 1860: 11. [1860?], non Jacq., Observ. Bot. 3: 1. 1768.

###### Type.

Unknown.

###### Notes.

As the original protologue for *E.laurifolia* (Todaro 1860) could not be found, it is not possible to determine if this name was validly published and thus illegitimate, as it was already validly published by Jacquin (1768), or it can be considered as a *nomen nudum*. Moreover, the protologue is cited on digital databases as being published in 1860, but according to TL-2 it was published either in 1859 or 1862. Todaro (1862) later published the variety E.crista-gallivar.laurifolia based on his previous *E.laurifolia*, but made no additional informative description that could help confirm the identity of the mentioned specimen.

##### ﻿ Erythrina crista-galli var. speciosa

Taxon classificationPlantaeFabalesFabaceae

Tod., Index. Sem. Panorm. 1861: 32. 1862

FD8DB302-091A-5F8D-8C3B-4EF9AC678A2D

≡ Erythrina speciosa Tod., Index. Sem. Panorm. 1860: 11. [1860?], non Lamb. ex Andrews, Bot. Repos. 7: tab. 443. 1807.

###### Type.

Unknown.

###### Notes.

As the original protologue for *E.speciosa* (Todaro 1860) could not be found, it is not possible to determine if this name was validly published and thus illegitimate, as it was already validly published by Andrews (1807), or it can be considered as a *nomen nudum*. Moreover, the protologue is cited on digital databases as being published in 1860, but according to TL-2 it was published either in 1859 or 1862. Todaro (1862) later published the variety E.crista-gallivar.speciosa based on his previous *E.speciosa*, but made no additional informative description that could help confirm the identity of the mentioned specimen.

##### ﻿ Erythrina crista-galli var. corallina

Taxon classificationPlantaeFabalesFabaceae

N.F.Mattos, Loefgrenia 71: 3. 1977

C60317B1-4BF9-59C8-A0AF-A506C6D670AE

###### Type.

Unknown.

###### Notes.

As the original protologue (Mattos 1977) could not be found, nor any collection made by Mattos was found in any herbaria, this variety could not be confirmed as a valid name or synonym.

##### ﻿3. Erythrina falcata

Taxon classificationPlantaeFabalesFabaceae

Benth., in Martius, Fl. Bras. 15(1): 172. 1859 nom. cons.

9D5AD550-2457-5841-8922-3FE03CA15BDC

Fig. 3

≡ Corallodendron falcatum (Benth.) Kuntze, Revis. Gen. Pl. 1: 172. 1891.
= Erythrina martii Colla, Herb. Pedem. 2: 250. 1834, nom. rej. Type: Brazil. Rio de Janeiro: “Campos [Campos dos Goytacazes?]”, s.d., s.leg., s.n. (lectotype, designated by Moraes et al. 2013, pg. 200: TO [1793, image seen]). (1)
= Erythrina crista-galli L. var. inermis Speg. & Girola, Anal. Soc. Rural Argent. 44: 335. 1910. Type: Argentina. Misiones: Puerto León, s.d., *Venturi 63* (lectotype, designated here: LIL [61285, image seen]; isotype: LP [presumably lost]). (2)

###### Type material.

**Brazil. Maranhão**: “in sylvis ad flumen Itapicurú prov. Maragnanensis”, s.d., *Martius s.n.* (lectotype, designated by Krukoff 1938, pg. 233 [first-step]; and Martins and Tozzi 2018, pg. 399 [second-step]: M [M0213337]; isolectotypes: M [M0213336, M0213338]). Residual syntypes: Brazil. Bahia: “in campis prov. Bahiensis australis”, s.d., *Wied-Neuwied s.n.*, BR (BR0000006584023); Minas Gerais: “in prov. Minarum ad Caxoeira do Campo”, 1839, *Claussen 119*, K (K000930965); “prope Barbacena”, s.d., *Saint-Hilaire 155* P (P00758901); *158*, P (P00758955); *159*, P (P00758954).

###### Notes.

**Figure 3.**
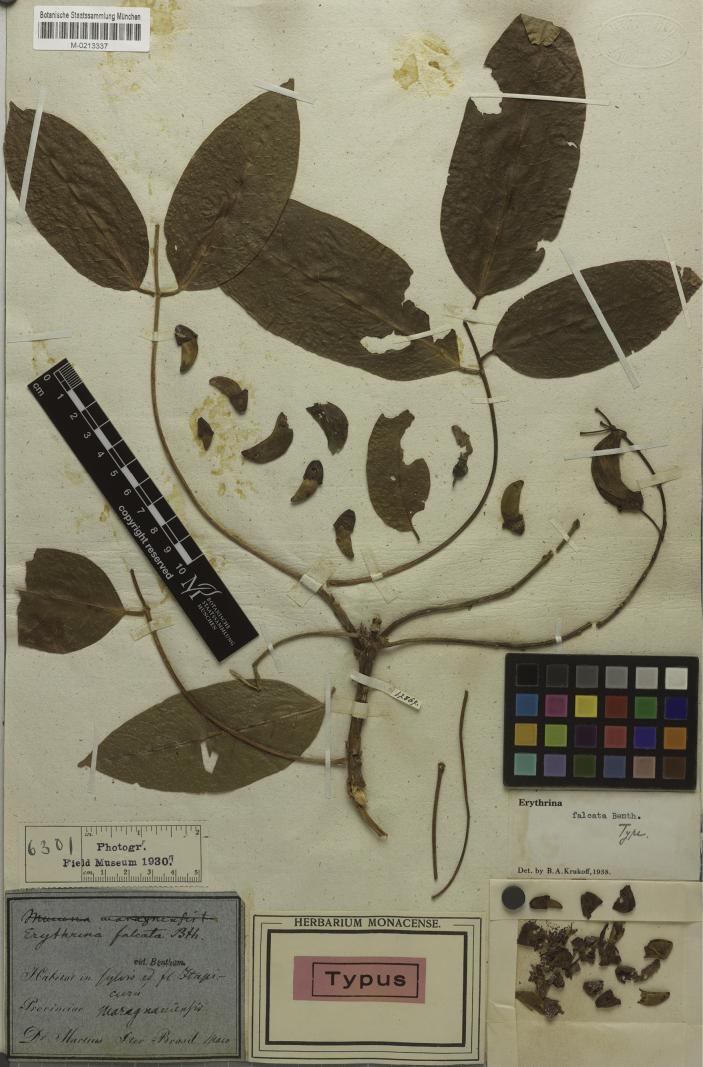
Lectotype of *Erythrinafalcata* Benth., in von Martius (1859: 172), designated by Krukoff (1938: 233, first-step) and Martins and Tozzi (2018: 399, second-step). Source: Botanische Staatssammlung München (M) via JSTOR Global Plants, M0213337.

Martius (1859) published *E.falcata* by Bentham mentioning four different collections from Brazil: one from the state of Maranhão (*Martius s.n.*), one from Bahia (*Wied-Neuwied s.n.*), and two from Minas Gerais (*Claussen s.n.* and *Saint-Hilaire s.n.*), but without mentioning any herbaria. Krukoff and Barneby (1974) accessed the collection by Martius in herbarium M and selected it as the type specimen. Because there are three different exsiccatae in the mentioned herbarium, Martins and Tozzi (2018) designated one of them as a second-step lectotype (Fig. 3). Despite not mentioning the herbaria of the other syntypes from Bahia and Minas Gerais, some collections are cited here with what was found in digital herbaria and believed to be the original ones. Kuntze (1891) published *Corallodendron* mentioning *E.falcata* as a synonym of *C.falcatum*, but the genus was later synonymized under *Erythrina* in Engler and Prantl (1894). Additional material: BR (BR0000013473808, photo n.v.), F (neg. 6301, negative of M0213337), IAN (IAN001758, photo of F neg. 6301), MO (MO-1680376, photo of F neg. 6301), P (P02951457, photo of M0213337).

(1) Colla (1834) published *E.martii* as a doubtful species from Brazil with a short description that resembles *E.falcata*, but without mentioning any collections. Krukoff (1938) already placed it as a doubtful synonym of *E.falcata*, but did not see the type specimen, which he believed could be at herbarium TO. Moraes et al. (2013), in a treatment of Brazilian plants distributed by Martius in 1827 and published by Colla in “*Herbarium Pedemontanum*” (Piedmont, Italy), confirmed the synonymy and designated a collection in herbarium TO, where the original material was deposited, as the lectotype of the name. We got access to an image of the TO collection and agree with the synonymy. Furthermore, as the name *E.martii* had priority over *E.falcata* due to its date of publication, as specified in Article 11 of the ICN (Turland et al. 2018), Martins and Tozzi (2015) proposed to conserve *E.falcata* since it was a very well-established name for the species. The proposal was approved by the Nomenclature Committee for Vascular Plants of the ICN at the XIX International Botanical Congress (Applequist 2016; Turland et al. 2017; Wilson 2017).

(2) Spegazzini and Girola (1910) published the variety E.crista-gallivar.inermis, which was doubtfully synonymized under *E.falcata* by Krukoff (1938), who did not see the type specimen. The synonymy was later confirmed by Lozano and Zapater (2010). Furthermore, Gutiérrez et al. (2002) investigated the type collections by Spegazzini in herbarium LP and found that only wood samples were collected for some species. As those samples could not be found anywhere in the LP collections, the holotype for this name is presumably lost. The protologue also could not be found online, but we had access to an image of the isotype’s exsiccata at LIL and designated it here as the lectotype.

###### Etymology.

The specific epithet “*falcata*” is derived from Latin, meaning “curved” or “sickle-shaped”, and was presumably chosen due to its corolla falcate shape, especially the standard and keel petals.

###### Vernacular names.

According to herbaria labels and Carvalho (2003), *E.falcata* is generally known as “mulungu” or “corticeira” in Brazil, and also “bico-de-papagaio”, “bico-de-pato”, “canivete”, “corticeiro-de-mato”, “marrequeira”, “mochoco” (and spelling variations), “moxoqueiro” (and spelling variations), “mulungu-coral”, “mutuqueiro” (and spelling variations), “pau-cebola”, “sanandú” (and spelling variations), “sananduí”, “sananduba” (and spelling variations), “sapato-de-judeu”, “suinã” (and spelling variations), “suinã-do-brejo” or “sumaúma” in the state of **Minas Gerais**; “bico-de-papagaio”, “canivete”, “coral”, “corticeira-ceboleiro”, “corticeira-da-serra”, “corticeiro-de-mato”, “marrequeira”, “mochoco” (and spelling variations), “mochoqueiro” (and spelling variations) or “letuíno” in **Paraná**; “canivete”, “mulungú tijolo”, “mulungú suinã”, “sanandú” (and spelling variations) or “sanandú do brejo” in **Rio de Janeiro**; “bituqueiro (and spelling variations), “camarão-assado”, “ceibo”, “corticeira-da-serra” or “corticeira-do-mato” in **Rio Grande do Sul**; “bico-de-papagaio”, “bituqueira” (and spelling variations), “corticeira”, “corticeira-da-serra”, “facãozinho”, “mituqueira” (and spelling variations) or “sinhanduva” in **Santa Catarina**; and “bico-de-arara”, “bico-de-papagaio”, “canivete”, “corticeira”, “feijão-bravo”, “machoco” (and spelling variations), “mulungu-coral”, “mutuqueiro” (and spelling variations), “sapatinho-de-judeu”, “sanandu” (and spelling variations), “sananduí”, “sananduva” (and spelling variations), “suinã” (and spelling variations), “suinã-da-mata”, “suinã-da-serra”, “suinã-mulambo” or “vermelheira” in **São Paulo**.

##### ﻿4. Erythrina fusca

Taxon classificationPlantaeFabalesFabaceae

Lour., Fl. Cochinch. 2: 427. 1790, based on “ Gelala Aquatica” Rumph., Herb. Amboin. 2: 235. 1741.

8B72711F-F208-5B46-90F6-0E4E8AEAA7DE

Fig. 4

≡ Corallodendron fuscum (Lour.) Kuntze, Revis. Gen. Pl. 1: 172. 1891.
= Erythrina glauca Willd., Neue Schriften Ges. Naturf. Freunde Berlin 3: 428. 1801. Type: Venezuela. Caracas: s.loc., s.d., *Hoffmannsegg s.n.* (lectotype, designated here: B [B-W13101-010]). (1)
≡ Duchassaingia glauca (Willd.) Walp., in Duchassaing and Walpers, Linnaea 23(=7): 742. 1851.
≡ Corallodendron glaucum (Willd.) Kuntze, Revis. Gen. Pl. 1: 172. 1891.
= Erythrina ovalifolia Roxb., Hort. Bengal.: 53. 1814, nom. nud.; Fl. Ind. 3: 254. 1832. Type: India. West Bengal: a scarce tree about Calcutta, s.d., s.leg., s.n. (lectotype, designated here: illustration in Wight 1839, tab. 247). (2)
≡ Duchassaingia ovalifolia (Roxb.) Walp., in Duchassaing and Walpers, Linnaea 23(=7): 742. 1851.
= Erythrina patens Moc. & Sessé ex DC., Prodr. 2: 414. 1825; A.DC., Calques Fl. Mexique 2: tab. 255. 1874. Type: [the Caribbean?]. s.loc., s.d., *Sessé et al. 3693* (lectotype, designated by Krukoff and Barneby 1974, pg. 340 [first-step]; and here [second-step]: MA [MA601534]; isolectotypes: MA [MA601535, MA601536]). (3)
≡ Corallodendron patens (Moc. & Sessé ex DC.) Kuntze, Revis. Gen. Pl. 1: 173. 1891.
= Erythrina caffra Blanco, Fl. Filip. 2: 394. 1845, nom. superf. et illeg., non Thunb., Prodr. Fl. Cap. 2: 121. 1800. Type: Philippines. s.loc., s.d., s.leg., s.n. (lectotype, designated by Martins and Tozzi 2018, pg. 399: illustration in Blanco et al. [1883?], tab. [526?]). (4)
= Erythrina ovalifolia Roxb. var. inermis Pulle, Nova Guinea 8(2): 651. 1912. Type: Indonesia. Western New Guinea: “am Noord-Fluss in einem verlassenem Dorfe”, 4 September 1909, *Römer 28* (holotype: L [L 0018975, sheet I; L 0018976, sheet II]). syn. nov. (5)
= Erythrina fusca Lour. var. inermis Rock, Legum. Pl. Hawaii: 188. 1920. Type: U.S.A. Hawaii, Honolulu: in cultivation on Anapuni Street, s.d., s.leg., s.n. (lectotype, designated here: illustration in Rock 1920, tab. 77). (6)

###### Type material.

**Indonesia.** “In Amboina raro occurrit. Arborescens in Lariqua & Hitoe, longa vero ſeu fruteſcens juxta ripas fluminis Elephantis, ubique non longe a mari. Magna vero copia reperitur in Java, Baleya, Borneo & Sumatra, uti & modicum in Ceramæ ora Orientali”, s.d., s.leg., s.n. (lectotype, designated by Martins and Tozzi 2018, pg. 399: illustration in Rumphius 1741, tab. 78).

###### Notes.

**Figure 4.**
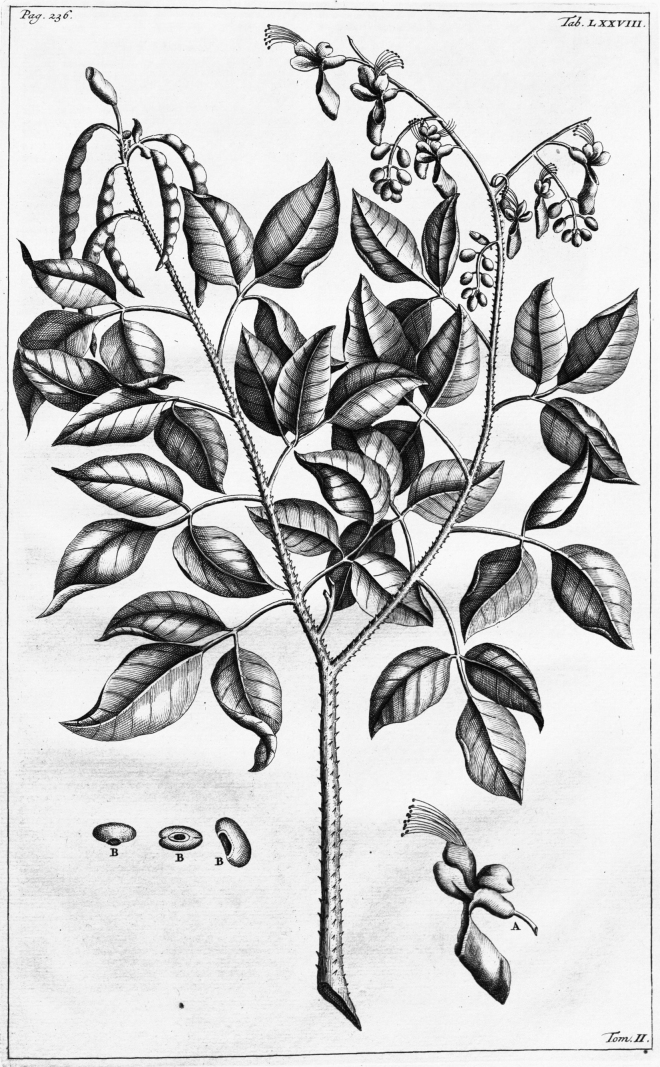
Lectotype of *Erythrinafusca* Lour. (1790: 427), designated by Martins and Tozzi (2018: 399). Source: Missouri Botanical Garden – Peter H. Raven Library via Biodiversity Heritage Library, available at https://www.biodiversitylibrary.org/page/187502.

Rumphius (1741) published descriptions and illustrations of three species from Ambon Island (Indonesia) that he called “*Gelala*”, before Linnaeus’ binomial system. Then, Loureiro (1790) published *E.fusca* from Vietnam mentioning the name “*Gelala Aquatica*” as a synonym, and Rumphius’ illustration was correctly designated by Martins and Tozzi (2018) as the lectotype of the name (Fig. 4). Kuntze (1891) published *Corallodendron* mentioning *E.fusca* as a synonym of *C.fuscum*, but the genus was later synonymized under *Erythrina* in Engler and Prantl (1894).

(1) Willdenow (1801) published *E.glauca* from Caracas (Venezuela), but did not mention any type specimen. A collection from Caracas labeled as *E.glauca* was found in Willdenow’s type specimens’ section in herbarium B with the same description given by him, and it was thus designated here as the lectotype. The name has been considered a synonym of *E.fusca* since Krukoff and Barneby (1974). Walpers (1851, not 1850) published *Duchassaingia* mentioning *E.glauca* as a synonym of *D.glauca*, but the genus was later synonymized under *Erythrina* in Engler and Prantl (1894). Kuntze (1891) published *Corallodendron*, mentioning *E.glauca* as a synonym of *C.glaucum*, but the genus was also synonymized into *Erythrina* in Engler and Prantl (1894). Additional material: F (neg. 2372, photo of B-W13101-010), IAN (IAN001757, photo of F neg. 2372).

(2) Roxburgh (1814) mentioned *E.ovalifolia* from India, but did not describe the species, so this name was first considered a *nomen nudum*. However, he fully described the species in Flora Indica (Roxburgh 1832), although no type specimen was assigned. Wight (1839, not 1840) published a redrawing of Roxburgh’s unpublished plates of species described in 1832, and his illustration for *E.ovalifolia* was designated here as the lectotype. The name has been considered a synonym of *E.fusca* since Krukoff and Barneby (1974). Walpers (1851, not 1850) published *Duchassaingia* mentioning *E.ovalifolia* as a synonym of *D.ovalifolia*, but the genus was later synonymized under *Erythrina* in Engler and Prantl (1894).

(3) De Candolle (1825) published *E.patens* based on a plate made by Sessé and Mociño for the Flora Mexicana, later published by Alph. De Candolle (De Candolle 1874). As stated by Krukoff and Barneby (1974), the species does not occur in Mexico and must have been collected somewhere in the Caribbean. The authors mentioned a collection by Sessé, Mociño, Castillo and Maldonado as the type, but did not mention any herbaria. Three exsiccatae of this collection were found in herbarium MA and one of them was designated here as the lectotype, in a second-step lectotypification. Kuntze (1891) published *Corallodendron* mentioning *E.patens* as a synonym of *C.patens*, but the genus was later synonymized under *Erythrina* in Engler and Prantl (1894).

(4) Blanco’s description (Blanco 1845) of *E.caffra* from the Philippines matches *E.fusca*, but as the name was already validly published by Thunberg (1800), Blanco’s publication was considered illegitimate. Martins and Tozzi (2018) correctly designated his illustration in Flora de Filipinas 3^rd^ edn. (Blanco et al. 1883?) as the lectotype, but according to TL-2 (Stafleu 1976), both its publication date and plate number remain doubtful.

(5) Pulle (1912) published the variety E.ovalifoliavar.inermis from Indonesia based only on the absence of spines, a character with well-documented morphological plasticity in *Erythrina* species (Guedes-Oliveira et al. manuscript in preparation). The exsiccatae found in herbarium L undoubtedly place the name as a synonym of *E.fusca*. The variety was already synonymized in Krukoff and Barneby (1974), but as the authors mistakenly cited it as “E.fuscaLour.varinermis”, the correct name is designated here as a new synonym.

(6) Rock (1920) published the variety E.fuscavar.inermis from a specimen being cultivated in Hawaii after seeds brought from Manila (the Philippines), based only on the absence of spines, which is a character with well-documented morphological plasticity in *Erythrina* species (Guedes-Oliveira et al. manuscript in preparation). His photograph was designated here as the lectotype of the name, which has been considered a synonym since Krukoff and Barneby (1974).

###### Etymology.

The specific epithet “*fusca*” is derived from Latin, meaning “dark” or “dusky”, and it was presumably chosen due to the dark-orange color of the petals in some individuals, described as “*fuſco-ruber*” in the protologue of the species. It is important to point out that the color of the petals varies a lot in this species, from shades of light-yellow to dark-orange and even vinaceous-red (Guedes-Oliveira et al. manuscript in preparation).

###### Vernacular names.

According to herbaria labels, *E.fusca* is generally known in Brazil as “mulungu”, and also as “alecrim” in the state of **Acre**; “açacurana” (and spelling variations) or “assacu branco” in **Amazonas**; “assacurana” (and spelling variations) in **Amapá**; “eritrina-da-baixa” or “sumaúma” in **Bahia**; “abobinha” or “flor-de-aboboreira” in **Mato Grosso**; “abobreiro” in **Mato Grosso do Sul**; “assacuhy”, “parica” or “pau angico” in **Pará**; and “assacurana” in **Rio de Janeiro**.

#### Doubtful synonyms of *Erythrinafusca*

##### ﻿ Erythrina picta

Taxon classificationPlantaeFabalesFabaceae

Blanco, Fl. Filip.: 565. 1837, nom. superf. et illeg., non L., Sp. Pl. 2: 993. 1763

B94F1F81-3F63-5493-B333-EC8A317D3B57

###### Type.

Unknown.

###### Notes.

Blanco (1837) published *E.picta* from the Philippines with a short description but without mentioning any type specimen. However, as Linnaeus (1763) had already validly published it before, it is an illegitimate name. The name was synonymized by Krukoff and Barneby (1974) as *E.fusca*, but could not be confirmed and thus remains here as a doubtful synonym.

##### ﻿ Erythrina atrosanguinea

Taxon classificationPlantaeFabalesFabaceae

Ridl., J. Straits Branch Roy. Asiat. Soc. 59: 93. 1911

3AECA1EF-CB35-5DD8-9F9B-31F29BE6D27F

###### Syntypes.

Malaysia. Kedah: Lankawi on the sea shore at Kwah; common round Alor Sta; Bukit Pinang, January 1897, *Ridley 15134; 15135*.

###### Notes.

Ridley (1911) published *E.atrosanguinea* from Malaysia with a full description and type specimens, but without mentioning any herbaria. Krukoff (1939) considered this a synonym of *E.fusca*, but he could not see the type specimens. As Ridley described its flowers as “deep red black”, and the type collections were not found on digital databases, it remains as a doubtful synonym.

##### ﻿5. Erythrina mulungu

Taxon classificationPlantaeFabalesFabaceae

Mart. ex Benth., in Martius, Fl. Bras. 15(1): 173. 1859.

A947AB34-3CB1-53C1-A17A-BCE79BCFAEB9

Fig. 5

≡ Corallodendron mulungu (Mart. ex Benth.) Kuntze, Revis. Gen. Pl. 1: 173. 1891.
= Erythrina chacoënsis Speg., in Spegazzini and Girola, Anal. Soc. Rural Argent. 44: 369. 1910; Lillo, Seg. Contr. Arb. Argent.: 20. 1924. Type: Argentina. Formosa, January 1883, *Venturi 281* (holotype: LP [LP010837]). (1)
= Erythrina dominguezii Hassl., Physis 6(21): 123. 1922. Type: Argentina. Formosa: “prope Guayculee”, September 1918, *Jörgensen 3215* (lectotype, designated by Lozano and Zapater 2010, pg. 188: BA [64115, image seen]; isolectotypes: BM [n.v.], G [GH00066286], LIL [n.v.], S [n.v.], US [US00004482]). Residual syntypes: Paraguay. San Pedro: “in silvis ripariis”, December 1916, *Rojas 2061*, BAF (BAF00000133), SI (SI002044, image seen; SI002045, image seen); Alto Paraguay: “Puerto Casado (flum. Paraguay), in silvis”, 1916, *Rojas 2122*, AS (n.v.). (2)
= Erythrina xinguensis Ducke, Arch. Jard. Bot. Rio de Janeiro 3: 167. 1922. Type: Brazil. Pará: “prope Altamira (Xingú), in silvis secundariis, terries argillosis compactis rufis fertilissimis”, 21 August 1919, *Ducke s.n.* (lectotype, designated here: RB [RB00540259!]; isotypes: B [presumably destroyed], F [V0059282F, frag. and photo of F neg. 2379], K [K000502768], NY [NY00008010, frag. slide, right envelope; NY00008018, frag., top envelope], R [R000043635!], RB [RB00547685!], S [S-R-9685], U [U.1243348]). syn. nov. (3)

###### Type material.

**Brazil. Minas Gerais**: “in silvis Catingas prope Contendas [Águas de Contendas] prov. Minarum”, s.d., *Martius 1582* (lectotype, designated by Krukoff 1938, pg. 241 [first-step]; and Martins and Tozzi 2018, pg. 399 [second-step]: M [M0240565]; isolectotype: M [M0240564]).

###### Notes.

**Figure 5.**
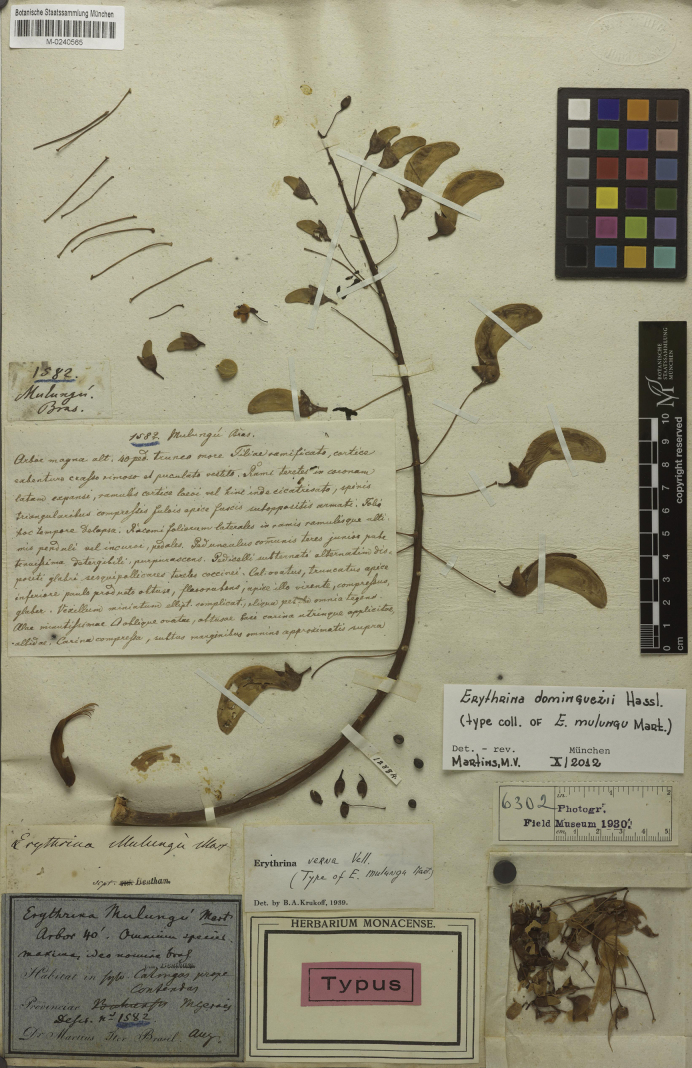
Lectotype of *Erythrinamulungu* Mart. ex Benth., in Martius (1859: 173), designated by Krukoff (1938: 241, first-step); and Martins and Tozzi (2018: 399, second-step). Source: Botanische Staatssammlung München (M) via JSTOR Global Plants, M0240565.

Bentham published a treatment of Brazilian *Erythrina* in Martius’ Flora Brasiliensis (Martius 1859). He included the name *E.mulungu*, given by Martius, citing a collection from the state of Minas Gerais but without mentioning any herbaria. Krukoff and Barneby (1974) located a collection by Martius in herbarium M and designated it as the type specimen. However, as there were two different exsiccatae in the mentioned herbarium, Martins and Tozzi (2018) correctly selected one of them as a second-step lectotypification (Fig. 5). Some databases give the authorship of the name only to Martius, but Bentham is the author of this species in Flora Brasiliensis. Kuntze (1891) published *Corallodendron* mentioning *E.mulungu* as a synonym of *C.mulungu*, but the genus was later synonymized under *Erythrina* in Engler and Prantl (1894). Additional material: F (neg. 6302, negative of M0240565), IAN001759 (photo of F neg. 6302).

(1) Spegazzini published *E. chacoënsis* in Spegazzini and Girola (1910), but the original protologue could not be found online. However, Lillo (1924 [reprint from 1917]) published Notas sobre el Herbario Venturi, mentioning the name and the original collection of Venturi in Argentina. An exsiccata from the same location was found at herbarium LP, which has the inscription “Erythrina chacoënsis Speg. (n. sp.)” and follows the description given by Lillo; hence it is believed to be the original material cited by Spegazzini. The name has been considered as a synonym of *E.dominguezii* since Krukoff (1938), who believed *E.mulungu* was a synonym of *E.verna* Vell., which was later found to be a different and accepted species by Martins and Tozzi (2018).

(2) Hassler (1922) published *E.dominguezii* mentioning three collections, one from Argentina (*Jörgensen 3215*) and two from Paraguay (*Rojas 2061* and *2122*), but without citing any herbaria. Lozano and Zapater (2010) then correctly designated the collection from Jörgensen as the lectotype of the name. The name was considered accepted until Martins and Tozzi (2018), who correctly synonymized it under *E.mulungu*. As for the remaining syntypes, some collections are cited here with what was found on digital herbaria and believed to be the original ones. Additional material: MO (MO-1624248, photo n.v.), P (P02934647, photo of GH00066286).

(3) Ducke (1922) published *E.xinguensis* from Brazil, with a full description and correctly citing the type specimen. However, as there were two exsiccatae in herbarium RB with the same number but without any information regarding a possible division of the same collection in different sheets (e.g. part 1 of 2), one of them was designated here as the lectotype. This name has been synonymized under *E.ulei* Harms since Krukoff (1938), and it caused much confusion in herbaria as the subsequent taxonomists could not properly identify the species. Ducke’s collections, in fact, correctly represent *E.mulungu* (Guedes-Oliveira et al. manuscript in preparation) and the name is thus synonymized here. Additional material: F (neg. 2379, negative of B), IAN001764 (photo of F neg. 2379), MO-1680465 (photo of F neg. 2379).

###### Etymology.

The specific epithet “*mulungu*” is derived from the most common vernacular name applied to all *Erythrina* species in Brazil. The origin is unknown, and it has many different meanings in African languages, most referring to a deity or a god creator of everything, father of all gods (e.g. Frankl 1990).

###### Vernacular names.

According to herbaria labels, *E.mulungu* is generally known as “mulungu” in Brazil, and also “pau-de-tiriça” in the state of **Minas Gerais**; “abobreira” (and spelling variations) or “maleitoso” in **Mato Grosso do Sul**; “açacurana” (and spelling variations) in **Pará**; and “bico-de-papagaio” in **São Paulo**.

##### ﻿6. Erythrina poeppigiana

Taxon classificationPlantaeFabalesFabaceae

(Walp.) O.F.Cook, Bull. Div. Bot. U.S.D.A. 25: 57. 1901.

18955848-2824-5149-BEBB-1E84C2D93F8E

Fig. 6

≡ Micropteryx poeppigiana Walp., in Duchassaing and Walpers, Linnaea 23(=7): 740. 1851.
≡ Erythrina micropteryx Poepp. ex Walp., in Duchassaing and Walpers, Linnaea 23(=7): 740. 1851, nom. nud.
≡ Erythrina micropteryx Poepp. ex Urb., Symb. Antill. 1: 327. 1899.
≡ Erythrina poeppigiana (Walp.) Skeels, in Galloway, Bull. Bur. Pl. Industr. U.S.D.A. 242: 84. 1912, nom. superf. et illeg., syn. nov.
= Erythrina amasisa Spruce, J. Proc. Linn. Soc., Bot. 3: 202. 1859. Type: Peru. San Martín: “Tarapoto, in sylvis montium inferiorum praecipue secus rivolus”, s.d., *Spruce 4069* (lectotype, designated by Krukoff 1938, pg. 237 [first-step]; and here [second-step]: K [K000200903, sheet I; K000200905, sheet II]; isolectotypes: BM [BM000778350], BR [BR0000005196685], E [E00296690], F [V0043470F, V0043471F], G [G00365292, sheet I; G00365293, sheet II], GH [GH00066283], K [K000200906, sheet I; K000200904, sheet II], MPU [MPU023232], NY [NY00007984, NY00007985], P [P00708429, P00708430], TCD [TCD0004415]. (1)
= Erythrina pisamo Posada-Ar., Estudios Cient.: 120. 1909. Type: Colombia. s.loc., s.d., s.leg., s.n. (lectotype, designated by Martins and Tozzi 2018, pg. 400: illustration in Molina 1909, tab. s.n.). (2)
= Erythrina darienensis Standl., Contr. U.S. Nat. Herb. 18: 108. 1916. Type: Panamá. Darién: near Boca de Pauarandó, on the Sambú River, southern Darién, February 1912, *Pittier 5578* (holotype: US [US00004481]; isotypes: BM [BM000931444], GH [GH00066265], MO [MO-114002, n.v.], NY [NY00007903, frag.; NY00007904]). (3)
= Erythrina poeppigiana (Walp.) O.F.Cook f. redmondii Steyerm. & Lasser, Phytologia 48: 286. 1981. Type: Veneuela. Miranda: “Los Chorros, Avenida principal, Caracas, en frente de la Escuela Hebraica”, 9 March 1981, *Redmond s.n.* (holotype: VEN [VEN137030]; isotypes: MO [MO-277140], NY [NY00008000, NY00008001], U [U0003538], US [US00153764]). syn. nov. (4)

###### Type material.

**Peru.** “Peruvia subandina. In sylvis”, September 1829, *Poeppig 1306* (lectotype, designated by Martins and Tozzi 2018, pg. 400: NY [NY00016336]; isolectotypes: B [presumably destroyed], F [V0043502F, frag. and photo of F neg. 2373; V0059555F, frag.?]).

###### Notes.

**Figure 6.**
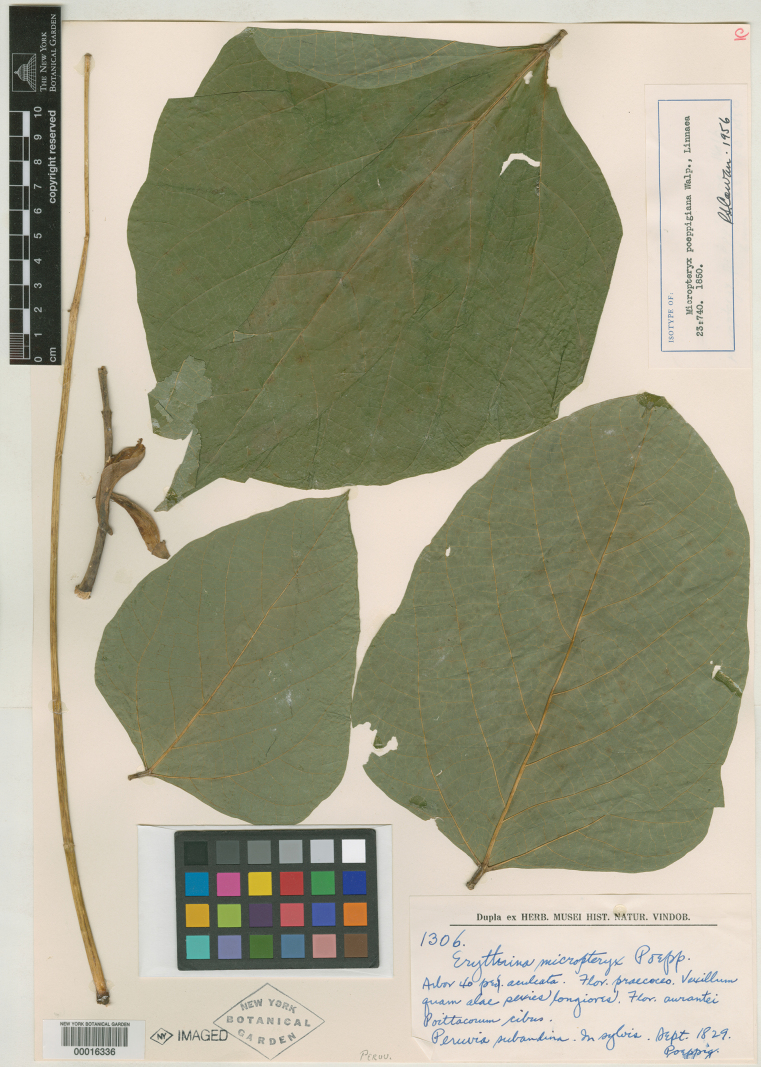
Lectotype of *Erythrinapoeppigiana* (Walpers) O.F.Cook (1901: 57), designated by Martins and Tozzi (2018: 400). Source: William and Lynda Steere Herbarium (NY) – The New York Botanical Garden via C. V. Starr Virtual Herbarium, NY00016336.

Walpers (1851, not 1850) published *Micropteryx* with a new species from Peru (*M.poeppigiana*), which was based on *Erythrinamicropteryx*, a name given by Poeppig that was never published. This mention also resulted in the publication of *E.micropteryx* itself, although it is considered a *nomen nudum*. Urban (1899) validly published the name *E.micropteryx* from Poeppig again. As the genus *Micropteryx* proposed by Walpers was later synonymized under *Erythrina* in Engler and Prantl (1894), Cook (1901) published *E.poeppigiana* based on *M.poeppigiana* by Walpers, which is the name that has been used for the species ever since. Krukoff (1938) designated the collection of Poeppig in herbarium B as the lectotype, but it was presumably destroyed in the bombing raid in 1943 (Botanischer Garten und Botanisches Museum Berlin 2022). Thus, Martins and Tozzi (2018) correctly designated the remaining available material in herbarium NY as the new lectotype (Fig. 6). Skeels (Galloway 1912) again published *E.poeppigiana* based on *M.poeppigiana* by Walpers, but as the name was already validly published by Cook (1901), it is considered illegitimate. Additional material: F (neg. 2373, negative of B), IAN001761 (photo of F neg. 2373), MO-1684973 (photo n.v.).

(1) Spruce (1859) published *E.amasisa* from Peru, but did not mention any type specimen. Krukoff (1938) cited a collection from Spruce in herbarium K as the type, but as there were three different exsiccatae in the mentioned herbarium, one of them was designated here as a second-step lectotypification.

(2) Posada-Arango (1909) published *E.pisamo* from Colombia but did not assign any type specimen. It was subsequently correctly designated by Martins and Tozzi (2018) as his illustration in the same publication. The name was already synonymized under *E.poeppigiana* by Krukoff (1938).

(3) Standley (1916) validly published *E.darienensis* from Panama with a full description mentioning the type specimen, and the name was already synonymized under *E.poeppigiana* by Krukoff (1938). Additional material: P (P02951340, photo of US00004481), W (W19390013217, photo of US00004481).

(4) Steyermarkii and Lasser (1981) published the form E.poeppigianaf.redmondii from Venezuela based only on some specimens with yellow flowers, and correctly cited the type specimen. As no other known characteristics support the distinction between forms (Guedes-Oliveira et al. manuscript in preparation), nor is this variation reported from elsewhere, it is designated here as a new synonym.

###### Etymology.

The specific epithet “*poeppigiana*” was a homage to Eduard Friedrich Poeppig (1798–1868), a German botanist, zoologist, and explorer who collected the type specimen attributed to the species.

###### Vernacular names.

There are no other known vernacular names for *E.poeppigiana* in Brazil besides the commonly used “mulungu”.

##### ﻿7. Erythrina similis

Taxon classificationPlantaeFabalesFabaceae

Krukoff, Brittonia 3: 271. 1938.

6B236688-9B00-5749-9902-2AE8D8133B33

Fig. 7

###### Type material.

**Paraguay. Central**: In the region of Lake Ypacaray, Februrary 1913, *Hassler 11450* (holotype: MO [MO-2050072]; isotypes: A [A00066288], BM [BM000538332], G [G00381487; G00381504, two sheets; G00381507, two sheets], K [K000502765], L [L0018977], NY [NY00008005, frag. slide, top left envelope; NY00008006], US [US00004504]).

###### Notes.

**Figure 7.**
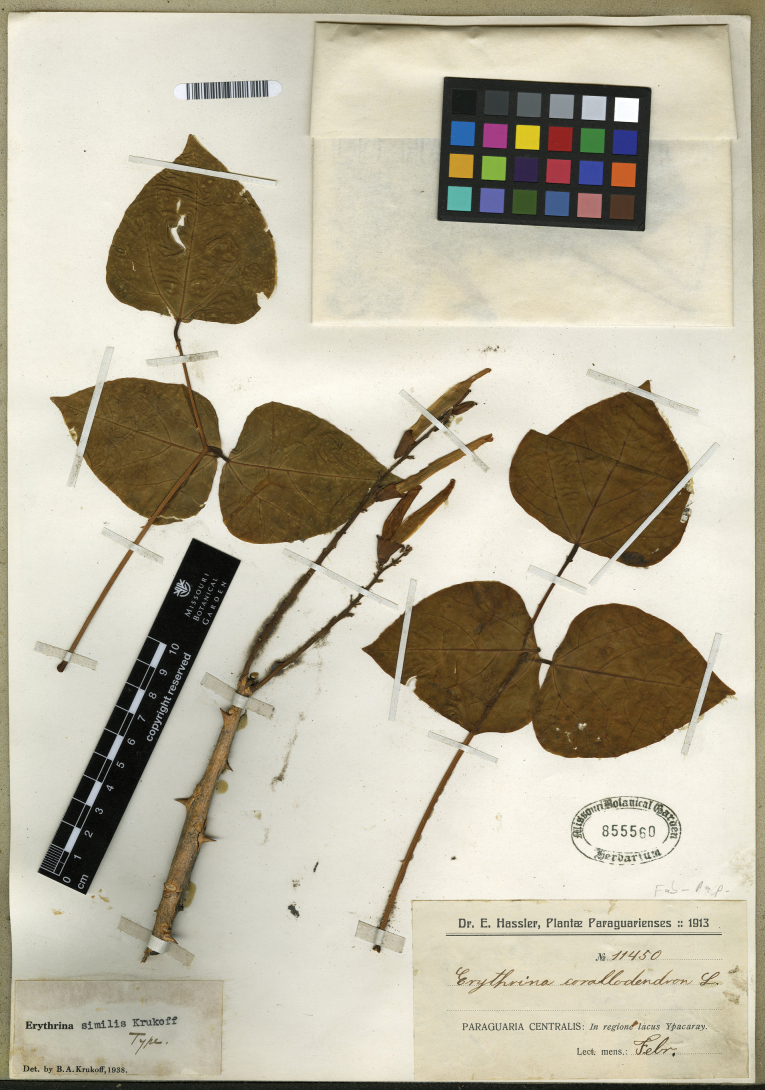
Holotype of *Erythrinasimilis*Krukoff (1938: 271). Source: Missouri Botanical Garden (MO) via Tropicos, MO-2050072.

There are no nomenclature issues with *E.similis*, as the name was validly published and the type was specimen correctly cited (Fig. 7). However, all databases and studies of the genus state that the protologue was published in 1939. Still, according to the journal’s website, the publication date is October 1938. Additional material: P (P02960062, photo of MO-2050072).

###### Etymology.

The specific epithet “*similis*” is derived from Latin, meaning “*similar to*”, and was chosen due to *E.similis* similarity with *E.amazonica*, especially in dried specimens.

###### Vernacular names.

There are no other known vernacular names for *E.similis* in Brazil besides the commonly used “mulungu”.

##### ﻿8. Erythrina speciosa

Taxon classificationPlantaeFabalesFabaceae

Lamb. ex Andrews, Bot. Repos. 7: tab. 443. 1807.

5F41D125-5F17-5940-9378-A1888A5819FE

Fig. 8

= Erythrina poianthes Brot. ex Tilloch & Taylor, Philos Mag. J. 61: 465. 1823; Brotero, Trans. Linn. Soc. Lond. 14: 342. 1824. Type: Portugal. “Colitur in Horto Botanico Olisiponensi [Jardim Botânico de Lisboa] ad Aulam Regiam in Ajuda sito, et alibi in Lusitania”, ex hort., s.d., s.leg., s.n. (lectotype, designated by Martins and Tozzi 2018, pg. 400: illustration in Brotero 1824, tab. 11). (1)
≡ Erythrina poianthes Brot., Trans. Linn. Soc. Lond. 14: 342. 1824, nom. superf. et. illeg.
≡ Stenotropis berteroi Hassk., Retzia 1: 183. 1855.
= Erythrina poianthes var. subinermis Lindl., Edwards’s Bot. Reg. 19: 1617. 1833. Type: England. London: Growing in the stove of his Grace the Duke of Northumberland at Sion, ex hort., s.d., s.leg., s.n. (lectotype, designated by Martins and Tozzi 2018, pg. 400: illustration in Lindley 1833, tab. 1617). (2)
= Erythrina reticulata C.Presl, Symb. Bot. 2: 22. 1834. Type: Brazil. Rio de Janeiro: “in sepibus”, ex hort., s.d., s.leg., s.n. (lectotype, designated by Martins and Tozzi 2018, pg. 400: illustration in Presl 1834, tab. 68). (3)
≡ Micropteryx reticulata (C.Presl) Walp., in Duchassaing and Walpers, Linnaea 23(=7): 741. 1851.
≡ Corallodendron reticulatum (C.Presl) Kuntze, Revis. Gen. Pl. 1: 173. 1891.
= Erythrina speciosa var. rosea N.F.Mattos, Loefgrenia 21: 1. 1967. Type: Brazil. São Paulo: Cultivada no Jardim Botânico de S. Paulo; proc.: Barra de Una, ao norte de Bertioga, na estrada Bertioga - S. Sebastião, ex hort., s.d., *Pires s.n.* (holotype: SP [SP000991]; isotype: RB [RB00514374!]). (4)

###### Type material.

**England.** s.loc., ex hort., s.d., s.leg., s.n. (lectotype, designated by Martins and Tozzi 2018, pg. 400: illustration in Andrews 1807, tab. 443).

###### Notes.

**Figure 8.**
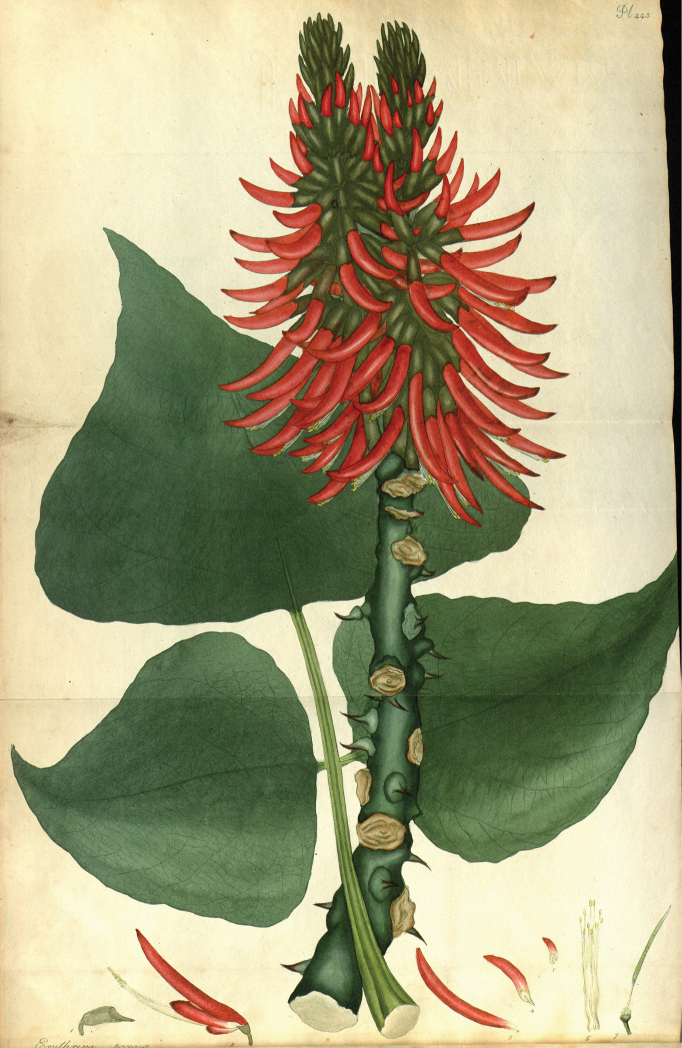
Lectotype of *Erythrinaspeciosa* Lamb. ex Andrews (1807: tab. 443), designated by Martins and Tozzi (2018: 400). Source: Missouri Botanical Garden – Peter H. Raven Library via Biodiversity Heritage Library, available at https://www.biodiversitylibrary.org/page/35501082.

Andrews (1807) published *E.speciosa*, a name communicated by Lambert from a cultivated specimen in British greenhouses, with a short description and a well-made illustration of a leaf, inflorescence, and flowers. As Andrews did not mention any type specimen, only citing that the species is “supposed to be a native of South America”, Martins and Tozzi (2018) correctly designated his illustration as the lectotype (Fig. 8).

(1) Brotero (1824) published *E.poianthes* from a cultivated specimen in the Jardim Botânico de Lisboa (Portugal), with a complete description and two very detailed illustrations of leaves, inflorescences, and dissected flowers, but without mentioning any type specimen. However, his description was published in the previous year by Tilloch and Taylor (1823) as a resumed version for the Proceedings of Learned Societies. This publication takes place due to the principles of priority as specified in Article 11 of the ICN (Turland et al. 2018), meaning the name published by Brotero is illegitimate. The name has been treated as a synonym since Krukoff (1938), and Martins and Tozzi (2018) correctly designated one of Brotero’s illustrations as the lectotype. Hasskarl (1855) published *Stenotropisberteroi* based on *E.poianthes* by Brotero, but the genus was later synonymized under *Erythrina* in Engler and Prantl (1894).

(2) Lindley (1833) published the variety E.poianthesvar.subnermis, with a short description and a well-made illustration of a leaflet and inflorescence from a cultivated specimen in a greenhouse in London (England). The variety was based solely on the absence of spines and the leafy habit in anthesis, which are characters with well-documented morphological plasticity in the species (Guedes-Oliveira et al. manuscript in preparation). The name was already synonymized under *E.speciosa* by Krukoff (1938). Lindley did not assign any type specimen, so Martins and Tozzi (2018) correctly designated his illustration as the lectotype for the name.

(3) Presl (1834) published *E.reticulata* from a specimen in Rio de Janeiro (Brazil), with a full description and a detailed illustration of leaves, inflorescence and dissected flowers, but without mentioning any type specimen. The name was already synonymized under *E.speciosa* by Krukoff (1938), but as he also did not designate any type specimen, Martins and Tozzi (2018) correctly designated Presl illustration as the lectotype. Walpers (1851, not 1850) published *Micropteryx* mentioning *E.reticulata* as a synonym of *M.reticulata*, but the genus was later synonymized into *Erythrina* in Engler and Prantl (1894). Kuntze (1891) published *Corallodendron* mentioning *E.reticulata* as a synonym of *C.reticulatum*, but the genus was synonymized under *Erythrina* in Engler and Prantl (1894) as well.

(4) Mattos (1967) published the variety E.speciosavar.rosea from a cultivated specimen in the Jardim Botânico de São Paulo (Brazil), based on the pinkish color of its flowers. The original protologue could not be found online, but the type specimens were seen. As there are no other morphological characters to support this variety besides the corolla’s color, which is a common mutation observed in cultivated specimens of *E.speciosa* (Guedes-Oliveira et al. manuscript in preparation), the name was already synonymized under *E.speciosa* by Martins and Tozzi (2018).

###### Etymology.

The specific epithet “*speciosa*” is derived from Latin, meaning “*handsome*” or “*splendid*”, and was presumably given by horticulturists who were amazed by the showy appearance of its flowers when the species was introduced to the United Kingdom.

###### Vernacular names.

According to Carvalho (2010) and herbarium records, *E.speciosa* is generally known as “mulungu” in Brazil, and also as “canivete”, “eritrina-anã”, “mulungu-da-várzea”, “mulungu-do-pequeno” or “suinã” (and spelling variations) in the state of **Minas Gerais**; “facãozinho”, “mulungu-do-litoral”, “suinã” (and spelling variations) or “unha-do-diabo” in **Paraná**; “bico-de-papagaio”, “candelabro-vermelho”, “corticeira”, “eritrina”, “eritrina-candelabro”, “mulungu-do-litoral”, “suinã” (and spelling variations) or “suinã-reticulata” in **São Paulo**, and “bico-de-papagaio” in **Santa Catarina**.

##### ﻿9. Erythrina ulei

Taxon classificationPlantaeFabalesFabaceae

Harms, in Ule, Verh. Bot. Vereins Prov. Brandenburg 48: 172. 1907.

B0A22137-5913-59F7-82A7-80A2979E3E08

Fig. 9

###### Type material.

**Peru. Loreto**: Yurimaguas, August 1902, *Ule 6300* (lectotype, designated here: K [K000502764]; isolectotypes: B [presumably destroyed], HBG [HBG519848], L [L0018978], NY [NY00008009, frag., bottom envelope; NY00521212, frag. slide, left envelope], RB [RB00540258!, mixture! fls. in packet are of *E.mulungu* Mart. ex Benth.]). Remaining syntype: Peru. Puno: “Provinz Sandia, Chunchusmayo, in der Nähe des Flusses”, 900 m, July 1902, *Weberbauer 1249*).

###### Notes.

**Figure 9.**
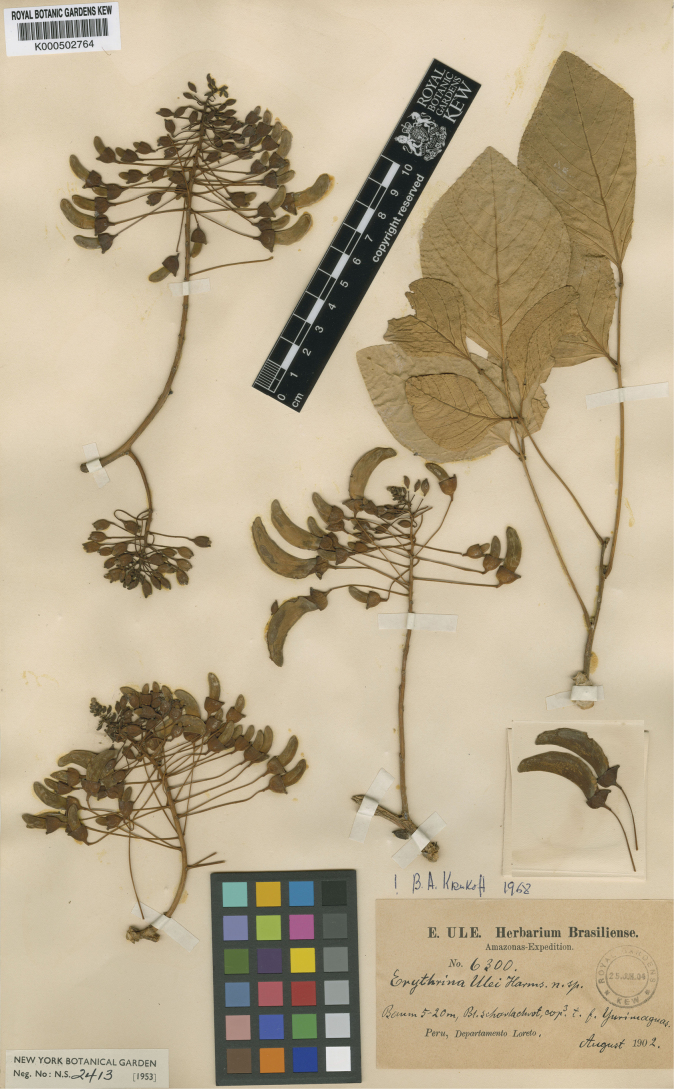
Lectotype of *Erythrinaulei* Harms, in Ule (1907: 172), designated here. Source: Kew Herbarium – Royal Botanic Gardens (K) via Kew Herbarium Catalogue, K000502764.

Ule (1907) published a complete description of a species named by Harms mentioning two different collections in Peru (*Ule 6300* and *Weberbauer 1249*), but without citing any herbaria. Krukoff (1938) correctly designated the collection from Ule in herbarium B as the lectotype, but it was presumably destroyed in the bombing raid in 1943 (Botanischer Garten und Botanisches Museum Berlin 2022). Therefore, here we select one of the remaining available specimens in herbarium K as the new lectotype (Fig. 9). The remaining syntype collection could not be found online. Additional material: F (neg. 2376, photo of B), IAN (IAN001760, photo of F neg. 2376), MG (MG006169, photo n.v.), MO (MO-1680466, photo n.v.; MO-1680467, photo n.v.).

###### Etymology.

The specific epithet “*ulei*” was a homage to Ernst Heinrich Georg Ule (1854–1915), a German botanist who collected one of the syntype specimens attributed to the species.

###### Vernacular names.

According to herbarium records, *E.ulei* is generally known as “mulungu” in Brazil, and also as “mandiocão” in the state of **Mato Grosso**.

##### ﻿10. Erythrina velutina

Taxon classificationPlantaeFabalesFabaceae

Willd., Neue Schriften Ges. Naturf. Freunde Berlin 3: 426. 1801.

604459CE-1F66-56BB-A086-A28672BD0C41

Fig. 10

≡ Chirocalyx velutinus (Willd.) Walp., Flora 36: 148. 1853.
≡ Corallodendron velutinum (Willd.) Kuntze, Revis. Gen. Pl. 1: 173. 1891.
= Erythrina aculeatissima Desf., Tabl. École Bot. 1: 191. 1804, nom. nud. Type: France. Île-de-France: “dans le jardin et dans les serres du Museum d’Histoire Naturelle”, ex hort., s.d., s.leg., s.n. (lectotype, designated by Krukoff and Barneby 1974, pg. 437: P [P02960024]). (1)
= Erythrina splendida Diels, Beitr. Veg. Ecuador: 96. 1937. Type: Ecuador. Guayas: Road from Guayaquil to Salinas, km. 89–90 from Guayaquil, just east of village of Buenos Aires. Alt. 35 m. Dry thorn scrub, 17 July 1986, *Plowman 14314* (neotype, designated here: F [V0448423F]). (2)
= Erythrina velutina f. aurantiaca (Ridl.) Krukoff, Brittonia 3: 329. 1938. Type: Brazil. Pernambuco: Main island, scattered bushes near the village and in the Sapate. One full-grown tree in the cocoa-nut plantation at Sueste, [1887?], *Ridley 35* (holotype: K [K000206207]; isolectotypes: BM [BM000931431], NY [NY00007988, frag. slide]). (3)
≡ Erythrina aurantiaca Ridl., J. Linn. Soc., Bot. 27: 30. 1890.

###### Type material.

**Venezuela. Distrito Capital**: Caracas, s.d., *Humboldt 653* (lectotype [not holotype], designated by Martins and Tozzi 2018, pg. 400: B [BW13100010, sheet I; BW13100020, sheet II; BW13100030, sheet III]; isolectotype: P [P00660125]).

###### Notes.

**Figure 10.**
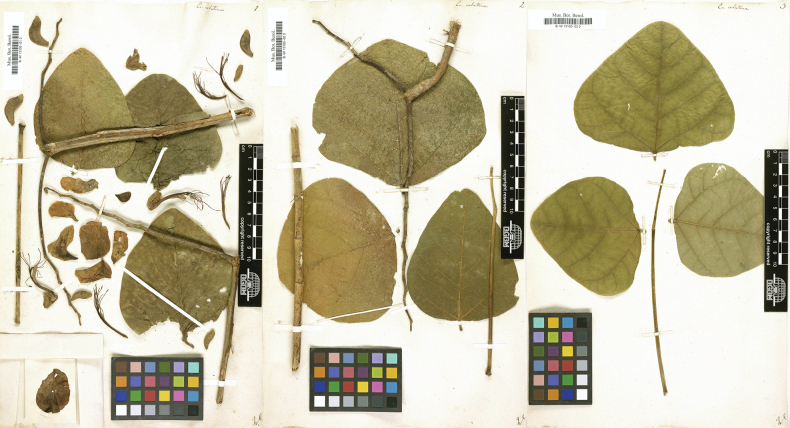
Lectotype of *Erythrinavelutina* Willd. (1801: 426), designated by Martins and Tozzi (2018: 400). Source: Herbarium Berolinense (B) – Botanic Garden and Botanical Museum Berlin, BW13100010, BW13100020, BW13100030.

Willdenow (1801) published *E.velutina* with a complete description of a specimen from Venezuela, but without mentioning any type specimen. Martins and Tozzi (2018) then designated, from the Willdenow’s type specimens in herbarium B, a collection from Humboldt composed of three sheets as the type of this name. However, as the authors mistakenly stated it as a holotype, the typification is corrected here to lectotype (Fig. 10). Walpers (1853, not 1854) published *Chirocalyx* mentioning *E.velutina* as a synonym of *C.velutinus*, but the genus was later synonymized under *Erythrina* in Engler and Prantl (1894). Kuntze (1891) published *Corallodendron* mentioning *E.velutina* as a synonym of *C.velutinum*, but the genus was also synonymized into *Erythrina* in Engler and Prantl (1894). Additional material: F (neg. 2378, photo of BW13100010), IAN (IAN001755, photo of F neg. 2378), MO (MO-1624337, photo n.v.; MO-1624338, photo n.v.).

(1) Desfontaines (1804) only mentioned the name *E.aculeatissima* in the Tableau de l’École de Botanique du Muséum d’Histoire Naturelle without giving any description, hence it is considered a *nomen nudum*. The name was considered doubtful by Krukoff (1938), but then was treated as a synonym of *E.velutina* by Krukoff and Barneby (1974), where the authors designated a collection in herbarium P as the type specimen.

(2) Diels (1937) published *E.splendida* from Ecuador mentioning a collection from the province of Guayas in herbarium B (*Diels 1230*) as the type specimen, which was presumably destroyed in the bombing raid in 1943 (Botanischer Garten und Botanisches Museum Berlin 2022). No duplicate or any other collection by Diels could be found on digital databases, so a collection from the same locality was designated as the neotype.

(3) Ridley (1890) published *E.aurantiaca* from a specimen in the archipelago of Fernando de Noronha (Pernambuco, Brazil), with a full description and an illustration of a leaf, inflorescence, dissected flowers, fruit and seeds. The name was later synonymized under E.velutinaf.aurantiaca by Krukoff (1938) based solely on a different coloring of the seeds of some specimens in the archipelago. As there were no other morphological characters to support this form, as the seeds vary in color both in the archipelago and on the mainland in Pernambuco and other Brazilian states (Guedes-Oliveira et al. manuscript in preparation), the name was synonymized into *E.velutina* by Martins and Tozzi (2018). Additional material: HUEFS (HUEFS000248863, photo of K000206207).

###### Etymology.

The specific epithet “*velutina*” is derived from Latin, meaning “*velvety*”, and was presumably chosen due to the abundance of trichomes in the species, especially on the petiole, abaxial leaflet surface, peduncle, pedicel, and calyx.

###### Vernacular names.

According to Carvalho (2008) and herbarium records, *E.velutina* is generally known as “mulungu” in Brazil, and also as “mulungu-do-ceará” in the state of **Amazonas**; “bucaré” or “mulungu-da-flor-amarela” in **Ceará**, where it is also the motive for the name of the municipality of Mulungu; “muchôco” or “mulungá” in **Minas Gerais**; and “mulungu-da-caatinga”, “pau-de-coral”, “sanandiú”, “sananduva” or “suinã” (and spelling variations) in **São Paulo**.

##### ﻿11. Erythrina verna

Taxon classificationPlantaeFabalesFabaceae

Vell., Fl. Flumin.: 304. 1829; Fl. Flumin. Icon. 7: tab. 102. 1831.

F57C51C0-35F2-5F55-BA72-683D549AB27B

Fig. 11

= Erythrina flammea Herzog, Repert. Spec. Nov. Regni Veg. 7: 57. 1909. Type: Bolivia. Santa Cruz: “Häufiger Baum in den Savannenwäldchen der Hügel von Buenavista”, ca. 400 m, October 1907, *Herzog 72* (holotype: Z [Z-000022779]). (1)

###### Type material.

**Brazil. Rio de Janeiro**: “Maritimis habitat”, s.d., s.leg., s.n. (lectotype, designated by Martins and Tozzi 2018, pg. 401: illustration in Biblioteca Nacional Digital Brasil [mss1198656_106]; also in Vellozo 1831, tab. 102); Brazil. Rio de Janeiro: Sta. Maria Magdalena, September 1913, *Constantino s.n.* (epitype, designated by Martins and Tozzi 2018, pg. 401: RB [RB00176986!]; isoepitypes: K [K000931001], NY [NY00600987], U [U.1243354, U.1243356], US [US02339391]).

###### Notes.

**Figure 11.**
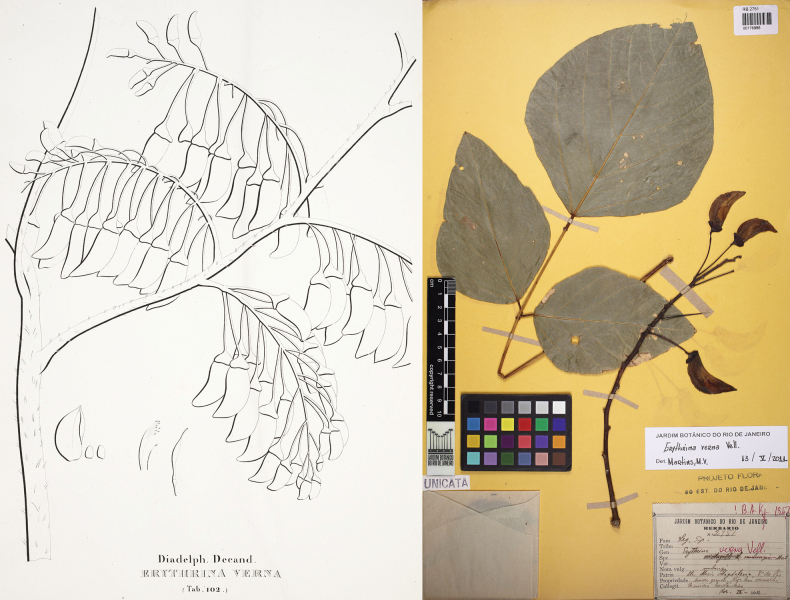
**Left**: Lectotype of *Erythrinaverna* Vell. (1829: 304; 1831: tab. 102), designated by Martins and Tozzi (2018: 401). Source: Biblioteca Nacional Digital Brasil - Fundação Biblioteca Nacional, available at http://objdigital.bn.br/acervo_digital/div_manuscritos/mss1198656/mss1198656_106.html; **Right**: Epitype of *E.verna* Vell., designated by Martins and Tozzi (2018: 401). Source: Dimitri Sucre Herbarium (RB) – Botanical Garden of Rio de Janeiro via Reflora Virtual Herbarium, RB00176986.

Vellozo (1829) published *E.verna* with a complete description, but without mentioning any type specimen. His illustration was published in Vellozo (1831), and Martins and Tozzi (2018) correctly designated it as the lectotype. However, as Vellozo’s illustration was an incomplete drawing of some inflorescences and dissected flowers that could be easily mistaken for some other *Erythrina* species, Martins and Tozzi (2018) also correctly designated an epitype to represent the species better (Fig. 11).

(1) Herzog (1909) validly published *E.flammea* from Bolivia with a complete description and mentioning the type specimen. The name was already synonymized under *E.verna* by Krukoff and Barneby (1974).

###### Etymology.

The specific epithet “*verna*” is derived from Latin, meaning “*related to spring*”, and it was presumably chosen due to the association of the flowering period of the species to the beginning of the spring season in the state of Rio de Janeiro, Brazil (September).

###### Vernacular names.

According to Carvalho (2014) and herbarium records, *E.verna* is generally known as “mulungu” in Brazil, and also as “corticeira” in the state of **Bahia**; “bico-de-papagaio”, “corticeira-ceboleiro”, “mulungu-de-flor-branca” or “suinã” (and spelling variations) in **Minas Gerais**; and “mulungú-de-várzea”, “suinã” or “suinã-da-Argentina” in **São Paulo**.

#### Hybrid species

##### ﻿ Erythrina × fluminensis

Taxon classificationPlantaeFabalesFabaceae

Barneby & Krukoff, in Krukoff and Barneby, Lloydia 37(3): 446. 1974.

7D33937C-21D5-5853-A9AD-902DC2D9C970

Fig. 12

###### Type material.

**Brazil. Rio de Janeiro**: “Guanabara, Horto Experimental do Aterro Glória-Flamengo; Culta de sementes recebidas de Bureau of Plant Introduction (U.S.A.). Cresceu no Horto do Museu Nacional e depois foi transplantada para o Aterro da Glória”, 26 August 1963, *Mello Filho 2025* (holotype: R [R000117879, two sheets]; isotype: NY [NY00007994]).

###### Notes.

**Figure 12.**
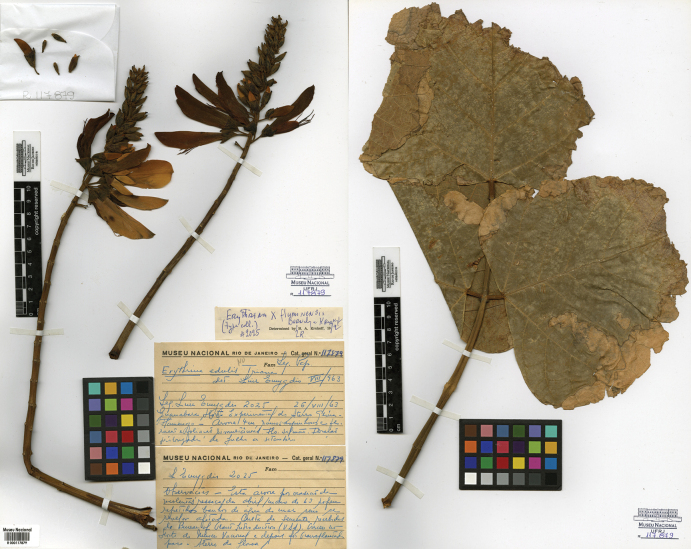
Holotype of *Erythrina×fluminensis* Barneby & Krukoff, in Krukoff and Barneby (1974: 446). Source: National Museum Herbarium (R) – Federal University of Rio de Janeiro via speciesLink, R000117879.

There are no nomenclature issues with E.×fluminensis, as the name was validly published, and the type specimen was correctly cited (Fig. 12). The species is a hybrid between *E.fusca* Lour. and *E.speciosa* Andrews (Guedes-Oliveira et al. manuscript in preparation).

###### Etymology.

The specific epithet “*fluminensis*” was based on the Portuguese word “fluminense”, which is derived from Latin meaning “inhabitant of river”. It was presumably chosen as a homage to the denomination of people born in the state of Rio de Janeiro, Brazil, where the hybrid species was first cultivated in the country and still exists to the day of this publication.

###### Vernacular names.

There are no other known vernacular names for E.×fluminensis in Brazil besides the commonly used “mulungu”.

#### Unplaced or excluded names

##### ﻿1. Erythrina velutina

Taxon classificationPlantaeFabalesFabaceae

Jacq., Pl. Hort. Schoenbr. 4: 34. 1804, auct., non Willd., Neue Schriften Ges. Naturf. Freunde Berlin 3: 426. 1801. nom. superf.

671C7CCC-DACC-5B79-8681-3B28855D7AA7

###### Notes.

Jacquin (1804) described *E.velutina* Willd. from a specimen cultivated in Schloss Schönbrunn (Austria), and this mention has been mistakenly considered as a new publication of this name. Moreover, his description matches *E.velutina* and even mentions Willdenow’s publication. However, the accompanying illustration represents a different species, so the name authored by Jacquin remains unplaced.

##### ﻿2. Erythrina secundiflora

Taxon classificationPlantaeFabalesFabaceae

Brot., Trans. Linn. Soc. Lond. 14: 346. 1824.

50712333-190E-5A95-BA64-0CC21A527D19

###### Type.

Portugal. Lisbon: “in Horto Regio Olisiponensi [Jardim Botânico de Lisboa], ad Aulam Regiam in Ajuda sitio, et alibi in Lusitania, ubi Martio Aprilique floret. Indigenam e Brasilia esse fertur”, ex hort., s.d., s.leg. s.n. (lectotype, designated here: illustration in Brotero 1824, tab. 12).

###### Notes.

Brotero (1824) published the species with a full description and a detailed illustration of leaves, inflorescence and dissected flowers. The description was based on a specimen cultivated at the Jardim Botânico de Lisboa and believed to have a native origin in Brazil. However, according to Krukoff (1938), the calyx as described and illustrated could indicate an association with the African *Erythrina* species, and not the American ones. Still, he pointed out that some characteristics depicted might be incorrect, so the name remains unplaced.

##### ﻿3. Erythrina nervosa

Taxon classificationPlantaeFabalesFabaceae

DC., Prodr. 2: 413. 1825. Type: Unknown.

3C0BA022-19BA-536F-91A3-210AEC818D07

≡ Corallodendron nervosum (DC.) Kuntze, Revis. Gen. Pl. 1: 173. 1891.

###### Notes.

The species was poorly described in De Candolle (1825) and remained as doubtful ever since its publication. Bentham in Martius (1859) believed it could be a native Brazilian species, but still maintained it as doubtful. According to Krukoff and Barneby (1974), this name is better placed as a synonym of *Callichlamyslatifolia* (A.Rich) K.Schum. (Bignoniaceae).

##### ﻿4. Erythrina corallodendrum

Taxon classificationPlantaeFabalesFabaceae

Vell., Fl. Flumin.: 304. 1829; Fl. Flumin Icon. 7: tab. 101. 1831, auct., non L., Sp. Pl. 2: 706. 1753.

426EE89B-459B-58AB-AFB0-58B9FF47FC38

###### Notes.

Vellozo (1829) included a species called “*E.corallodendrum*” in his treatment of Brazilian *Erythrina* in Florae Fluminensis, with a description and an illustration published later in Vellozo (1831), but without mentioning any type specimen. This citation was then considered to be a record of the occurrence of *E.corallodendrum* L. in Brazil rather than a new publication of the same name. Hence, there is no record of this name in any digital databases. However, Lima (1995) mentioned this name as being authored by Vellozo and placed it as a synonym of *E.speciosa*. The description and illustration given by Vellozo undoubtedly depict the unique morphological features of *E.speciosa*, but according to Recommendation 50D of Chapter VI of the ICN (Turland et al. 2018), “misidentifications should not be included in synonymies but added after them”, so we exclude the name here.

##### ﻿5. Erythrina mediterranea

Taxon classificationPlantaeFabalesFabaceae

Vell., Fl. Flumin.: 305. 1829; Fl. Flumin Icon. 7: tab. 103. 1831.

EEDC5DAF-FF06-5954-B1FB-01F25166E6CB

###### Type.

Brazil. São Paulo: “silvis mediterraneis transalpinis prope praedium Boavista”, s.d., s.leg. s.n. (lectotype, designated here: illustration in Biblioteca Nacional Digital Brasil [mss1198656_107]; also in Vellozo 1831, tab. 103).

###### Notes.

The species was poorly described in Vellozo (1829) and the accompanying illustration in Vellozo (1831) depicts a sterile branch from a specimen growing in the municipality of Cunha, state of São Paulo (Pastore et al. 2021). According to Krukoff (1938), it could represent either *E.crista-galli* L. or *E.falcata* Benth. As it is impossible to even confirm it as *Erythrina*, the name remains unplaced.

##### ﻿6. Erythrina adansonii

Taxon classificationPlantaeFabalesFabaceae

hort. ex Colla, Herb. Pedem. 2: 249. 1834. nom. nud.

D3DDE10A-6C40-5B98-AEB2-3A41AC747EAD

###### Notes.

Colla (1834) mentioned *E.adansonii* Hortul as a doubtful synonym of *E.crista-galli* L. However, as no other valid publication or description of this name was found elsewhere, it was excluded here as a *nomen nudum*.

##### ﻿7. Erythrina argentea

Taxon classificationPlantaeFabalesFabaceae

Blume ex Miq., Fl. Ned. Ind. 1(1): 207. 1855. nom. nud.

E30D85C5-09AD-598A-AD70-A3FA14658CE8

###### Notes.

Miquel (1855) mentioned *E.argentea* by Blume as synonym of *E.ovalifolia* Roxb. without any further information. As there was no other valid publication of this name elsewhere, it was excluded here as a *nomen nudum*.

##### ﻿8. Erythrina compacta

Taxon classificationPlantaeFabalesFabaceae

hort. ex W.Bull, Cat. New Beautiful Rare Pl.: 4. 1871, nom. nud.; Carrière and André, Rev. Hortic.: 348. 1882, nom. nud.

E15F47D5-E796-508B-A665-D90E892AE944

≡ Erythrina compacta W.Bull ex K.Koch, Wochenschr. Vereines Beförd. Gartenbaues Konigl. Preuss. Staaten 14(20): 159. 1871, nom. nud.

###### Notes.

Bull (1871) briefly described the aesthetic characteristics of a cultivated plant named *E.compacta* by horticulturists. Later in the same year, Koch (1871) mentioned the specimen cited by Bull, but only repeated his description. It was later also described by Carrière and André (1882), who observed that the specimen was just a more compact cultivated variety of *E.crista-galli*. According to Article 38 of the ICN (Turland et al. 2018), a name cannot be effectively published using a description of purely aesthetic features, which is the case for all three publications related to this name, thus all of them are excluded here as *nomen nudum*.

##### ﻿9. Erythrina fusca Lour. var. inermis

Taxon classificationPlantaeFabalesFabaceae

Pulle, Nova Guinea 8(2): 651. 1912, auct., non E. ovalifolia Roxb. var. inermis Pulle, Nova Guinea 8(2): 651. 1912.

629FD479-F6DB-50D8-82A4-5947E3CBE2BE

###### Notes.

Krukoff and Barneby (1974) mentioned “E.fuscavar.inermis” as a synonym of *E.fusca*, resulting in the publication of this name. However, as Pulle (1912) did not base his variety on *E.fusca* Lour. but on *E.ovalifolia* Roxb., the name is excluded here as misapplied.

##### ﻿10. Erythrina moelebei

Taxon classificationPlantaeFabalesFabaceae

Vieill. ex Guillaumin & Beauvis., Ann. Soc. Bot. Lyon 38: 87. 1914, nom. nud.

3E3594A8-1615-5206-986B-3E9BC2E3D3F0

###### Notes.

Guillaumin and Beauvisage (1914) cited *E.moelebei* by Vieillard in a list of species from New Caledonia, but without giving any additional information. As there was no description of this name elsewhere and the type specimen cited (*Vieillard 60*) could not be found, the name remains unplaced. Krukoff (1939) treated it as a “hyponym” of *E.fusca* Lour. without seeing the type specimen as well.

##### ﻿11. Erythrina dariensis

Taxon classificationPlantaeFabalesFabaceae

Standl., Contr. U.S. Nat. Herb. 18: 108. 1916, auct., non E. darienensis Standl., Contr. U.S. Nat. Herb. 18: 108. 1916.

06F59561-B849-5255-A4ED-1653BBE761A1

###### Notes.

Perkins and Payne (1970) misspelled *E.darienensis* Standl. as “*E.dariensis*”, which resulted in the publication of this name as well, so it is excluded here as misapplied.

##### ﻿12. Erythrina indica

Taxon classificationPlantaeFabalesFabaceae

(sensu R.Vig.), Arch. Bot. Mém. 6. [1944?], auct., in Du Puy et al., Legum. Madagascar: 516. 2002, non Lam., Encycl. 2: 391. 1786.

3949BC8F-7A10-59FC-950F-6862FEF8F6C5

###### Notes.

This name was mentioned by Du Puy et al. (2002) as a synonym of *E.fusca* Lour. in their treatment of *Erythrina* in The Leguminosae of Madagascar. The protologue is cited by the authors as being published in 1944, but according to BHP these publications by Viguier ranged only from 1927 to 1936. Moreover, some databases state that this name was used to refer to *E.fusca* Lour. As the original protologue could not be found, it remains unplaced.

## ﻿Conclusions

The present work highlights the importance of comprehensive and detailed work regarding scientific nomenclature, showing that there are still many issues to address even in a relatively well-known genus like *Erythrina*. Despite the treatments proposed by Krukoff (1938) and Krukoff and Barneby (1974), and the more recent works by Martins (2014) and Martins and Tozzi (2018), in this revision it was possible to designate several new synonyms and type specimens for names related to Brazilian *Erythrina*. We also highlighted the knowledge gaps that remain and should be addressed in future works. Moreover, the ever-growing importance of virtual herbaria, online databases and digital libraries with up-to-date and reliable scientific information is noteworthy mentioning. Virtually all type specimens and original protologues analyzed in this work were accessed via digital resources, which must remain available to any user through any electronic device with a reasonable internet connection.

## Supplementary Material

XML Treatment for Erythrina

XML Treatment for Erythrina amazonica

XML Treatment for Erythrina crista-galli

XML Treatment for Erythrina crista-galli var. laurifolia

XML Treatment for Erythrina crista-galli var. speciosa

XML Treatment for Erythrina crista-galli var. corallina

XML Treatment for Erythrina falcata

XML Treatment for Erythrina fusca

XML Treatment for Erythrina picta

XML Treatment for Erythrina atrosanguinea

XML Treatment for Erythrina mulungu

XML Treatment for Erythrina poeppigiana

XML Treatment for Erythrina similis

XML Treatment for Erythrina speciosa

XML Treatment for Erythrina ulei

XML Treatment for Erythrina velutina

XML Treatment for Erythrina verna

XML Treatment for Erythrina × fluminensis

XML Treatment for Erythrina velutina

XML Treatment for Erythrina secundiflora

XML Treatment for Erythrina nervosa

XML Treatment for Erythrina corallodendrum

XML Treatment for Erythrina mediterranea

XML Treatment for Erythrina adansonii

XML Treatment for Erythrina argentea

XML Treatment for Erythrina compacta

XML Treatment for Erythrina fusca Lour. var. inermis

XML Treatment for Erythrina moelebei

XML Treatment for Erythrina dariensis

XML Treatment for Erythrina indica

## ﻿Additional information

### Author ORCIDs

Ramon Guedes-Oliveira https://orcid.org/0000-0001-8122-0782

Ana Paula Fortuna-Perez https://orcid.org/0000-0003-4977-4341

Leandro Cardoso Pederneiras https://orcid.org/0000-0003-1822-227X

Vidal de Freitas Mansano https://orcid.org/0000-0002-7204-0744

### Data availability

All of the data that support the findings of this study are available in the main text or Supplementary Information.

## Supplementary materials

Supplementary material 1 *Erythrina* L. of Brazil: nomenclatural revision Data type xlsx Explanation note Binomials concerning the 11 accepted Brazilian species of *Erythrina* L., chronologically ordered, with protologue information (year: page or illustration), current status, and references (year: page) related to them. See text in Guedes-Oliveira et al. (2023) for details about authorships, typifications and synonymies. In bold the currently accepted names. https://binary.pensoft.net/file/900996This dataset is made available under the Open Database License (http://opendatacommons.org/licenses/odbl/1.0/). The Open Database License (ODbL) is a license agreement intended to allow users to freely share, modify, and use this Dataset while maintaining this same freedom for others, provided that the original source and author(s) are credited.Ramon Guedes-Oliveira, Ana Paula Fortuna-Perez, Leandro Cardoso Pederneiras, Vidal de Freitas Mansano
